# A novel application of 2-silylated 1,3-dithiolanes for the synthesis of aryl/hetaryl-substituted ethenes and dibenzofulvenes

**DOI:** 10.3762/bjoc.13.185

**Published:** 2017-09-08

**Authors:** Grzegorz Mlostoń, Paulina Pipiak, Róża Hamera-Fałdyga, Heinz Heimgartner

**Affiliations:** 1 Department of Organic and Applied Chemistry, University of Łódź, Tamka 12, PL 91-403 Łódź, Poland; 2 Department of Chemistry, University of Zürich, Winterthurerstrasse 190, CH-8057 Zürich, Switzerland

**Keywords:** diazomethanes, 1,3-dipolar cycloadditions, olefination reactions, reaction mechanisms, thioketones

## Abstract

Trimethylsilyldiazomethane (TMS-CHN_2_) reacts readily with hetaryl thioketones to give sterically crowded 2-trimethylsilyl-4,4,5,5-tetrahetaryl-1,3-dithiolanes with complete regioselectivity at −75 °C as well as at rt. Thiofluorenone, a relatively stable and highly reactive aryl thioketone, yields upon treatment with TMS-CHN_2_ at −60 °C the corresponding 1,3,4-thiadiazoline. This unstable cycloadduct undergoes decomposition at ca. −45 °C and the silylated thiocarbonyl *S*-methanide generated thereby is trapped with complete regioselectivity by aryl or hetaryl thioketones forming also sterically crowded 2-trimethylsilyl-1,3-dithiolanes. The obtained 1,3-dithiolanes, by treatment with an equimolar amount of TBAF in a one-pot procedure, are converted in high yields into hetaryl/aryl-substituted ethenes or dibenzofulvenes, respectively, via a cycloreversion reaction of the intermediate 1,3-dithiolane carbanion. The presented protocol offers a new, highly efficient approach to tetrasubstituted ethenes and dibenzofulvenes bearing aryl and/or hetaryl substituents.

## Introduction

Aryl and hetaryl-substituted ethenes form an important class of organic compounds with a growing number of applications in materials chemistry, crystal engineering, photooptics, etc. Among these, the thiophen-2-yl-substituted ethenes [1–5] as well as dibenzofulvenes-containing compounds [6–9] are of special interest. The development of methods for the synthesis of substituted ethenes is of great importance and the so-called olefination reactions allow for valuable functional-group transformations [10–13]. As a general method for the preparation of tetrasubstituted ethenes, the McMurry reaction is widely applied [14]. Another approach, which opens access to diverse ethenes, is the ‘two-fold extrusion reaction’, which comprises the [3 + 2]-cycloaddition of a diazo compound with a thiocarbonyl dipolarophile and subsequent elimination of N_2_ followed by sulfur extrusion [15–17].

**Scheme 1:**
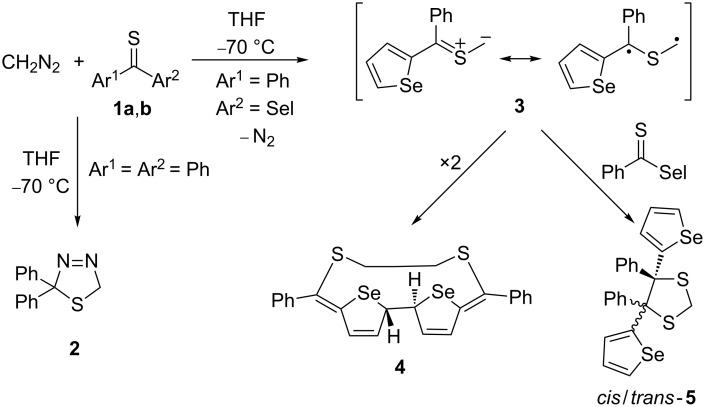
Reactions of diphenyl and phenyl selenophen-2-yl thioketones with diazomethane (CH_2_N_2_; Sel = selenophen-2-yl).

In our continuing studies on [3 + 2]-cycloadditions with thioketones and diazo compounds, we turned our attention to hetaryl thioketones [18]. It turned out that the presence of the hetaryl groups strongly influences the reactivity of these dipolarophiles in reactions with diazomethane (CH_2_N_2_, Schönberg reaction) [19–21]. For example, in contrast to thiobenzophenone (**1a**), phenyl selenophen-2-yl thioketone (**1b**) does not form the corresponding 2,5-dihydro-1,3,4-thiadiazole of type **2**, and even at −70 °C spontaneous elimination of N_2_ was observed. As products of this reaction, dimer **4** of the intermediate thiocarbonyl *S-*methanide **3** and the sterically crowded 4,4,5,5-tetraaryl-1,3-dithiolane **5** were obtained (Scheme 1) [20]. The formation of both products was rationalized by the assumption that the in situ formed **3a** reacts as a delocalized diradical species.

**Scheme 2:**
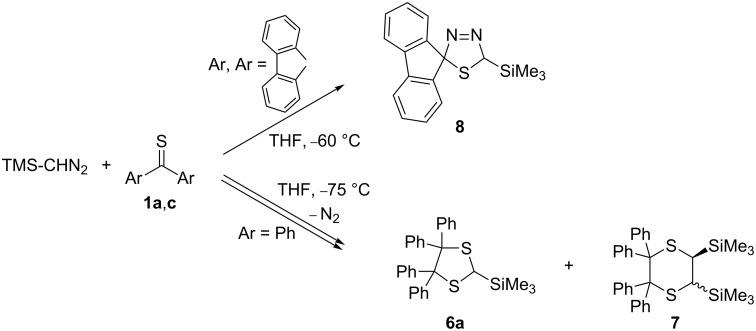
Reaction of diaryl thioketones with trimethylsilyldiazomethane (TMS-CHN_2_).

In a recent publication, similar reactions of TMS-CHN_2_ with **1a** and some dihetaryl thioketones, e.g., **1d** and **1e**, were reported [22]. In contrast to CH_2_N_2_, TMS-CHN_2_ reacted with thiobenzophenone (**1a**) with evolution of N_2_ even at −75 °C and led to a mixture of 4,4,5,5-tetraphenyl-1,3-dithiolane **6a** and 2,2,3,3-tetraphenyl-1,4-dithiane **7** (Scheme 2). This result demonstrated that TMS-CHN_2_ does not form a stable [3 + 2]-cycloadduct with **1a**, but, in analogy to **1b** in the reaction with CH_2_N_2_, spontaneous elimination of N_2_ takes place. In another study, we described a different behaviour of thiofluorenone (**1c**), which reacted with TMS-CHN_2_ at −60 °C to yield the expected [3 + 2]-cycloadduct **8**, which only at ca. −40 °C extruded N_2_ [23].

The goal of the present study was the preparation of a series of 2-trimethylsilyl-1,3-dithiolanes of type **6**, which, after desilylation, should be applied for nucleophilic additions of the 1,3-dithiolane anion with some electrophilic agents. An analogous sequence of reactions was described earlier for 2-aryl-2-trimethylsilyl-1,3-dithiolanes [24–25].

## Results and Discussion

The earlier described protocol for the reaction of thiobenzophenone (**1a**) with TMS-CHN_2_ [22] has been slightly modified and the reaction was performed at −70 °C using the reagents in a ratio of 3:1. Under these conditions, 1,3-dithiolane **6a** was formed almost exclusively with only traces of 1,4-dithiane **7** as revealed by ^1^H NMR analysis of the crude reaction mixture. Desilylation of **6a** occurred quantitatively and the known tetraphenylethylene (**9a**) [26] was obtained in 90% yield.

**Scheme 3:**
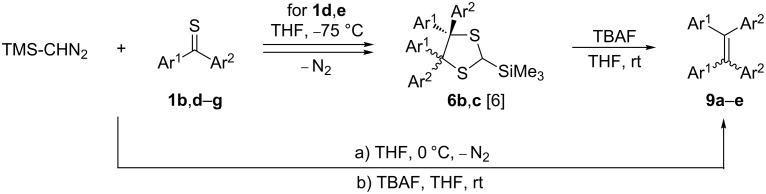
Formation of tetraaryl/hetarylethenes **9** from the reaction of TMS-CHN_2_ with diaryl/hetaryl thioketones **1** (for Ar^1^, Ar^2^ see Table 1).

Two symmetric dihetaryl thioketones, **1d** and **1e**, were reacted with TMS-CHN_2_ in THF at −75 °C, and after the spontaneous elimination of N_2_, the sterically crowded 4,4,5,5-tetrahetaryl-1,3-dithiolanes **6b** and **6c**, respectively, were obtained as exclusive products in high yields [22]. The isolated pure products were treated with equimolar amounts of TBAF in THF at room temperature and the progress of the reaction was monitored by TLC. After ca. 1 h, the crude reaction mixtures obtained after aqueous work-up were analysed by ^1^H NMR spectroscopy. Unexpectedly, in both cases, only signals of hetaryl rings were observed. The ^13^C NMR spectra allowed to identify both products as tetrasubstituted ethenes **9b**,**c** (Scheme 3, Table 1). The signals for the ethene C=C atoms appeared at 127.7 and 131.2 ppm, respectively. In addition, the melting point determined for **9b** confirmed its identity with the known tetrakis(thiophen-2-yl)ethene [1].

The same products **9b**,**c** were obtained in reactions at 0 °C in comparable yields, when the initially formed 1,3-dithiolanes **6b**,**c**, without isolation, were treated with equimolar amounts of TBAF. Using this protocol, the non-symmetrical thioketones **1b**,**f**,**g** were smoothly converted into the corresponding tetraarylethenes **9d**–**f** (Table 1). However, in these cases, mixtures of (*Z*)- and (*E*)-isomers were formed and isolated in 65–75% yield. In all cases, the ratio of isomers was calculated to ca. 3:2 (^1^H NMR); however, the attempted chromatographic separation was unsuccessful.

**Scheme 4:**
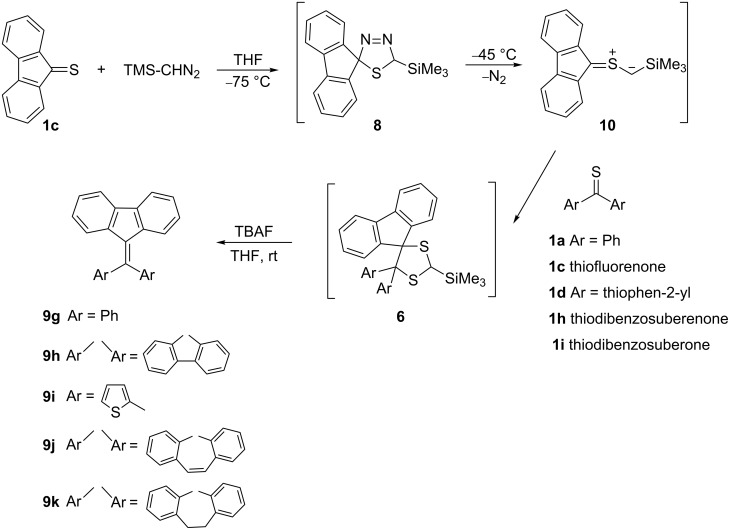
Synthesis of dibenzofulvenes **9g**–**k**.

The results obtained with thiofluorenone (**1c**) prompted us to prepare other 2-trimethylsilylated 1,3-dithiolanes of type **6**, available via [3 + 2]-cycloaddition of the in situ generated (at ca. −45 °C) silylated thiocarbonyl *S*-methanide **10** with thioketones **1a**,**c,d**,**h** and **i**. The obtained products of type **6**, without isolation, were desilylated at rt to give the expected ethenes **9g**–**k** with a fluorenylidene moiety (Scheme 4, Table 1).

**Table 1:** Synthesis of tetraaryl/hetarylethenes **9a−f** and dibenzofulvenes **9g−k** from diaryl thioketones **1** and TMS-CHN_2_.^a^

**1**	Ar^1^	Ar^2^	**6**	Ar^1^	Ar^2^	**9**	Ar^1^	Ar^2^	Yield [%]^b^

**a**	Ph	Ph				**a**	Ph	Ph	90
**b**	Ph	Sel	–			**f**	Ph	Sel	71
**d**	Thi	Thi	**b** [8]	Thi	Thi	**b**	Thi	Thi	89
**e**	Sel	Sel	**c** [8]	Sel	Sel	**c**	Sel	Sel	87
**f**	Sel	Fur	–			**d**	Sel	Fur	65
**g**	Ph	Thi	–			**e**	Ph	Thi	75
**c**	fluorenylidene		–			**g**	Ph	Ph	72
**c**	fluorenylidene		–			**h**	fluorenylidene		70
**c**	fluorenylidene		–			**i**	Thi	Thi	77
**c**	fluorenylidene		–			**j**	dibenzosuberenylidene		66
**c**	fluorenylidene		–			**k**	dibenzosuberylidene		62

^a^Ph = Phenyl, Thi = thiophen-2-yl, Sel = selenophen-2-yl, Fur = furan-2-yl. ^b^Yield of isolated ethenes **9** from the one-pot reaction.

**Scheme 5:**
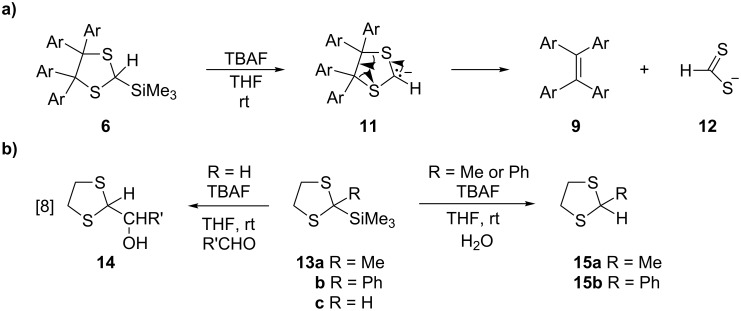
a) Mechanistic explanation for formation of ethenes **9** from dithiolanes of type **6** and b) desilylation reactions of 2-trimethylsilyl-1,3-dithiolanes **13**.

The unexpected formation of tetrasubstituted ethenes **9** from 1,3-dithiolanes of type **6** requires a mechanistic explanation. The behaviour of these dithiolanes under desilylation conditions differs significantly from that of 4,5-unsubstituted 1,3-dithiolane **13c** (Scheme 5). In the latter case, the desilylation leads to the corresponding carbanion, which can be trapped with an aldehyde to give **14** [24–25]. In contrast, the carbanion **11** undergoes a spontaneous cycloreversion to yield tetraaryl/hetarylethene **9** and dithioformic acid anion (**12**).

For comparison reasons, two other 2-trimethylsilyl 1,3-dithiolanes **13a**,**b** with no substituents at C4 and C5 were prepared and tested in desilylation reactions performed with TBAF in THF solutions. In both cases, quenching of the intermediate carbanions with water led to the corresponding 1,3-dithiolanes **15a** and **15b**, and no [3 + 2]-cycloreversion of the heterocyclic ring was observed (Scheme 5). As mentioned before, 1,3-dithiolane **13c** has been reported as a convenient source of a reactive carbanion, which subsequently was trapped with aromatic or aliphatic aldehydes as electrophilic agents yielding alcohols **14**, which should be considered as protected forms of corresponding α-hydroxyaldehydes [24–25]. Also in this series, no [3 + 2]-cycloreversion of the intermediate carbanion leading to the destruction of the heterocyclic ring was observed. These experiments demonstrate that the 4,5-unsubstituted 1,3-dithiolane carbanions generated under mild conditions through desilylation of the appropriate precursors **13** do not require an electron-withdrawing substituent for their stabilization [29].

The preparation of 1,3-dithianes and 1,3-dithiolanes, as well as the generation of the corresponding carbanions are widely applied in the umpolung chemistry [27–28] and in the chemistry of protective groups [29–30], but tetrasubstituted ethenes have never been prepared by using this approach.

## Conclusion

The presented study showed that 2-trimethylsilyl-4,4,5,5-tetraaryl-1,3-dithiolanes, readily available by treatment of hetaryl thioketones with trimethylsilyl diazomethane (TMS-CHN_2_), are superior substrates for the preparation of tetraarylethenes. Another group of 2-trimethylsilylated 1,3-dithiolanes, obtained through the [3 + 2]-cycloaddition of trimethylsilylated thiofluorenone *S*-methanide with aryl and hetaryl thioketones can also be used for this transformation. The described protocol opens a new, straightforward access to a series of new dibenzofulvenes [7] containing hetaryl rings, which can be of great interest in materials chemistry and related sciences. The key intermediate in the reaction is the 1,3-dithiolane carbanion, which, in contrast to the 4,5-unsubstituted analogues, undergoes a spontaneous cycloreversion reaction to give tetraarylethene and dithioformate anion. This method supplements the list of preparatively useful olefination reactions and is another proof for the utility of aryl and hetaryl thioketones in organic synthesis [18]. Another important feature of the presented system with a potential, practical application to materials and coordination chemistry is the release of strictly controlled amounts of the dithioformate anion under mild, neutral conditions [31].

## Experimental

**General information:** Solvents and chemicals were purchased and used as received without further purification. Products were purified by standard column chromatography on silica gel (230–400 mesh, Merck). Unless stated otherwise, yields refer to analytically pure samples. NMR spectra were recorded with a Bruker Avance III 600 MHz (^1^H NMR [600 MHz]; ^13^C NMR [151 MHz]) instrument. Chemical shifts are reported relative to solvent residual peaks (^1^H NMR: δ 7.26 ppm [CHCl_3_]; ^13^C NMR: δ 77.0 ppm [CDCl_3_]). IR spectra were registered with a FTIR NEXUS spectrometer (as film or KBr pellets). High resolution MS measurements were performed with a GCT Premier Waters instrument. Melting points were determined in capillaries with a Stuart SMP30 apparatus with automatic temperature monitoring. Microwave-supported syntheses of thioketones **1** were performed using the CEM-focused Microwave-type Discover SPD reactor.

**Starting materials:** Trimethylsilyldiazomethane (TMS-CHN_2_) was a commercial reagent which was used in all reactions as 1 M THF solution without further purification. Thiobenzophenone (**1a**), thiodibenzosuberenone (**1h**), and thiodibenzosuberone (**1i**) were prepared from the corresponding ketones by treatment with Lawesson’s reagent (L.R.) in toluene upon irradiation with microwaves (150 W) over 2 min [32]. The most efficient method for the preparation of thiofluorenone (**1c**) was the thionation of fluorenone by simultaneous passing of dry hydrogen chloride and hydrogen sulfide streams through the ethanolic solution at 0–5 °C (ice bath cooling) [33]. In analogy to **1a**, hetaryl thioketones **1b**,**d**–**g** were prepared from the corresponding ketones [34] by treatment with L.R. in toluene solution upon irradiation with microwaves over 2 min [32].

**General Procedure for the one-pot synthesis of ethenes 9a**–**f:** A hetaryl thioketone **1** (1 mmol) was dissolved in THF (2–3 mL) and the solution was cooled to 0 °C (ice bath). Then, the mixture was treated with small portions of an ethereal solution of TMS-CHN_2_ (2 M, 0.5 mL, 1 mmol). The reaction was complete after ca. 15 min (TLC, petroleum ether/CH_2_Cl_2_ 8:2). Then, a solution of TBAF (1 M in THF, 1 mL, 1 mmol) was added. After complete reaction (TLC, petroleum ether/CH_2_Cl_2_ 8:2), the solvent was removed under vacuum, and the residue was subjected to ^1^H NMR analysis in CDCl_3_ solution. The crude products **9a**–**f** were purified by column chromatography (Et_2_O/CH_2_Cl_2_ 8:2).

**1,1,2,2-Tetrakis(selenophen-2-yl)ethene** (**9c**): Yield: 236 mg (87%); chromatographic purification (petroleum ether/CHCl_3_ 8:2). Orange crystals; mp 199**–**202 °C; IR (KBr) ν: 3085 (w), 1445 (m), 1429 (m), 1236 (s), 1182 (m), 1116 (m), 1024 (m), 849 (m), 840 (m), 748 (s), 687 (s) cm^−1^; ^1^H NMR (600 MHz, CDCl_3_) δ 7.14**–**7.15 (m, 4 H_arom_), 7.20**–**7.21 (m, 4 H_arom_), 8.09 (dd, *J* = 6.0 Hz, 1.2 Hz, 4 H_arom_) ppm; ^13^C NMR (150 MHz, CDCl_3_) δ 129.1, 133.0, 134.2 (12 CH_arom_), 131.2 (2 C=), 149.6 (4 C_arom_) ppm; anal. calcd for C_18_H_12_Se_4_ (544.13): C, 39.73; H, 2.22; found: C, 39.68; H, 2.25.

**1,2-Diphenyl-1,2-bis(selenophen-2-yl)ethene** (**9f**, mixture of *E*/*Z* isomers, ratio 2:1.2). Yield: 156 mg (71%); chromatographic purification (petroleum ether/CHCl_3_ 8:2). Yellow crystals; mp 221**–**223 °C; IR (KBr) ν: 3044 (w), 1483 (w), 1439 (m), 1233 (m), 1201 (w), 1071 (w), 1021 (w), 770 (m), 706 (s), 685 (s) cm^−1^; ^1^H NMR (600 MHz, CDCl_3_) δ 6.53 (d, *J* = 3.6 Hz, 2 H_arom_), 6.96 (dd, *J* = 6.0 Hz, 2.4 Hz, 2 H_arom_), 7.06 (d, *J* = 3.6 Hz, 2 H_arom_), 7.11**–**7.15 (m, 12 H_arom_), 7.45**–**7.50 (m, 10 H_arom_), 7.80 (d, *J* = 6.0 Hz, 2 H_arom_), 8.03 (d, *J* = 5.4 Hz, 2 H_arom_) ppm; ^13^C NMR (150 MHz, CDCl_3_) δ 126.8, 127.6, 128.3, 128.4, 129.1, 129.4, 130.8, 131.4, 132.2, 132.6, 133.0, 133.1 (32 CH_arom_), 135.0, 136.5, 141.5, 142.9, 151.0, 151.7 (8 C_arom_, 4 C=) ppm; anal. calcd for C_22_H_16_Se_2_ (438.28): C, 60.29; H, 3.68; found: C, 60.09; H, 3.67.

**Synthesis of dibenzofulvenes 9g**–**k:** Thiofluorenone (**1c**, 98 mg, 0.5 mmol) was dissolved in THF (1.5 mL) and the solution was cooled to −78 °C (dry ice/acetone). Then, the mixture was treated with small portions of an ethereal solution of TMS-CHN_2_ (2 M, 0.25 mL, 0.5 mmol) until the intense colour of **1c** vanished. Next, the mixture was allowed to warm slowly to −45 °C, whereby elimination of N_2_ was observed. Then, a solution of thiofluorenone (**1c**) or di(thiophen-2-yl) thioketone (**1d**, 0.5 mmol) in 1.5 mL of THF was added. After ca. 10 min, the cooling bath was replaced by an ice bath (0 °C) and a solution of TBAF (1 M in THF, 1 mL, 1 mmol) was added to the mixture. After removal of the solvent under vacuum, the residue was subjected to ^1^H NMR analysis in CDCl_3_ solution. The crude products were purified by column chromatography.

**2,2’-[(9*H*-Fluoren-9-ylidene)methylene]dithiophene** (**9i**): Yield: 132 mg (77%); chromatographic purification (petroleum ether/CHCl_3_ 7:3). Orange crystals; mp 175**–**177 °C; IR (KBr) ν: 3094 (w), 3066(w), 1556 (w), 1445 (m), 1416 (m), 1252 (w), 1220 (w), 837 (w), 777 (m), 713 (s) cm^−1^; ^1^H NMR (600 MHz, CDCl_3_) δ 6.68**–**6.94 (m, 4 H_arom_), 7.02**–**7.04 (m, 2 H_arom_), 7.14**–**7.18 (m, 4 H_arom_), 7.42**–**7.43 (m, 2 H_arom_), 7.59**–**7.61 (m, 2 H_arom_) ppm; ^13^C NMR (150 MHz, CDCl_3_) δ 119.3, 124.8, 126.6, 127.4, 128.1, 128.5, 129.2 (14 CH_arom_), 137.7, 138.4, 140.6, 144.6 (6 C_arom_, 2 C=) ppm; HRMS–ESI^+^ (TOF): [M]^+^ calcd for C_22_H_14_S_2_, 342.0537; found, 342.0527.

**Preparation of 1,3-dithiolanes 13a**,**b**: Both compounds were prepared from commercial methyl (trimethylsilyl) ketone or phenyl (trimethylsilyl) ketone [35], respectively, by treatment with 1,2-ethanedithiol in the presence of BF_3_·Et_2_O following a literature protocol [36].

**2-Phenyl-2-(trimethyl)silyl-1,3-dithiolane** (**13b**): Yield: 191 mg (75%); colourless solid; mp 52**–**53 °C; IR (KBr) ν: 2952 (m), 2920 (m), 1591 (m), 1496 (m), 1480 (m), 1435 (m), 1245 (vs), 1078 (m), 1036 (m), 922 (m), 840 (vs), 736 (s), 698 (vs), 609 (m), 504 (s) cm^–1^; ^1^H NMR (600 MHz, CDCl_3_) δ 0.10 (s, 9H), 2.99–3.05 (m, 2H), 3.20–3.25 (m, 2H), 7.12–7.15 (m, 1H_arom_), 7.23–7.26 (m, 2H_arom_), 7.65–7.68 (m, 2H_arom_) ppm; ^13^C NMR (150 MHz, CDCl_3_) δ 2.1 (3 CH_3_), 38.9 (2 CH_2_), 58.7 (C(2)), 125.7, 127.4, 127.8 (5 CH_arom_), 144.6 (1 C_arom_) ppm; anal. calcd for C_12_H_18_S_2_Si (254.49): C, 56.63; H, 7.13; S, 25.20; found: C, 56.64; H, 7.37; S, 25.31.

**Desilylation of 1,3-dithiolanes 13a**,**b**. General procedure: The corresponding 1,3-dithiolane (0.5 mmol) was dissolved in THF (2 mL) and the solution was cooled to 0 °C (ice bath). Then, a solution of TBAF (0.5 mL, 1 M in THF, 0.5 mmol) was added portion-wise. The progress of the desilylation was controlled by TLC (petroleum ether/CH_2_Cl_2_ 7:3). After removal of the solvent under vacuum, the residue was subjected to ^1^H NMR analysis in CDCl_3_ solution. The crude products were purified by column chromatography (petroleum ether/CH_2_Cl_2_, increasing polarity to CH_2_Cl_2_).

## Supporting Information

File 1 Experimental data for compounds **9**, **13**, **15** and copies of the original ^1^H and ^13^C NMR spectra.
