# Supplementary material for: A novel application of 2-silylated 1,3-dithiolanes for the synthesis of aryl/hetaryl-substituted ethenes and dibenzofulvenes
Source: Beilstein J Org Chem. 2017 Sep 8;13:1900–6. doi: 10.3762/bjoc.13.185 (PMC5629374; doi:10.3762/bjoc.13.185)
Supplement: File 1 — Experimental data for compounds 9, 13, 15 and copies of the original 1H and 13C NMR spectra. [file Beilstein_J_Org_Chem-13-1900-s001.pdf]

Supporting Information

for

A novel application of 2-silylated 1,3-dithiolanes for the synthesis of aryl/hetaryl-substituted ethenes and dibenzofulvenes

Grzegorz Mlostoń*[1], Paulina Pipiak[1], Róża Hamera-Fałdyga[1] and Heinz Heimgartner[2]

Address:[1]Department of Organic and Applied Chemistry, University of Łódź, Tamka 12, PL 91-403 Łódź, Poland and [2]Department of Chemistry, University of Zürich, Winterthurerstrasse 190, CH-8057 Zürich, Switzerland

Email: Grzegorz Mlostoń - gmloston@uni.lodz.pl

*Corresponding author

Dedicated to Professor Tadeusz Marek Krygowski (Warsaw) on the occasion of his 80th birthday

Experimental data for compounds 9, 13, 15 and copies of the original ^{1}H and ^{13}C NMR spectra

1. Experimental data for compounds **9**, **13** and **15**.

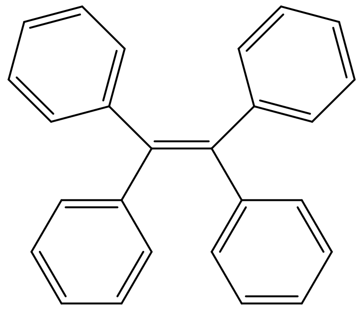

1,1,2,2-Tetraphenylethene (**9a**):[S1] Yield: 298 mg (90%); chromatografic purification (petroleum ether/CH_2Cl_2 7:3). White crystals; m.p. 222–224 °C (ref.[S1]: 219−221 °C). ^{1}H NMR (600 MHz, $CDCl_3$): δ = 7.10–7.14 (m, 12 H_{arom}), 7.04–7.08 (m, 8 H_{arom}), ppm. ^{13}C NMR (150 MHz, $CDCl_3$): δ = 143.7 (1 signal for 4 C_{arom}), 141.0 (1 signal for 2 C=), 131.3, 127.6, 126.4 (3 signals for 20 CH_{arom}) ppm.

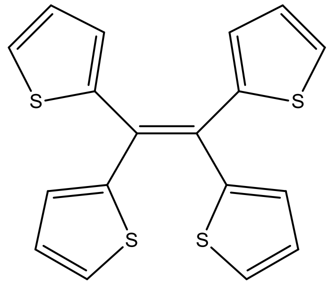

1,1,2,2-Tetrakis(thiophene-2-yl)ethene (**9b**):[S2] Yield: 158 mg (89%); chromatografic purification (petroleum ether/CH_2Cl_2 8:2). Yellow crystals; m.p. 193–195 °C (ref.[S2]: 196–198 °C). ^{1}H NMR (600 MHz, $CDCl_3$): δ = 7.31–7.32 (m, 4 H_{arom}), 6.94–6.95 (m, 4 H_{arom}), 6.88–6.89 (m, 4 H_{arom}) ppm. ^{13}C NMR (150 MHz, $CDCl_3$): δ = 144.2 (4 C_{arom}), 127.7 (2 C=), 129.9, 127.6, 126.6, (12 CH_{arom}) ppm.

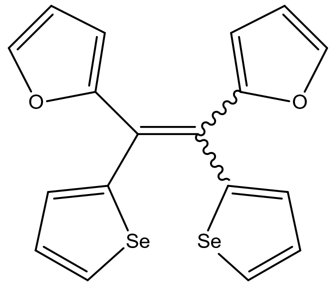

1,2-Bis(furan-2-yl)-1,2-bis(selenophen-2-yl)ethene (**9d**, mixture of *E*/*Z* isomers, ratio 1:0.7): Yield: 135 mg (65%); chromatografic purification (petroleum ether/$CHCl_3$ 8:2). Yellow crystals; m.p. 128–130 °C. IR (KBr): ν = 3098 (w), 3053 (w), 1480 (m), 1429

(m), 1251 (m), 1216 (m), 1154 (m), 1144 (m), 1074 (m), 1011 (s), 924 (m), 884 (m), 811 (m), 739 (s), 687 (s) cm^{-1}. ^1H NMR (600 MHz, $CDCl_3$): δ = 8.06 (dd, J = 5.4 Hz, 1.2 Hz, 4 H_{arom}), 7.39–7.40 (m, 2 H_{arom}), 7.35–7.36 (m, 2 H_{arom}), 7.15–7.20 (m, 6 H_{arom}), 7.04 (dd, J = 4.2 Hz, 1.2 Hz, 2 H_{arom}), 6.44 (m, 2 H_{arom}), 6.40–6.41 (m, 2 H_{arom}), 6.38 (d, J = 3.6 Hz, 2 H_{arom}), 6.17 (d, J = 3.6 Hz, 2 H_{arom}) ppm. ^{13}C NMR (150 MHz, $CDCl_3$): δ = 153.4, 154.2, 149.5, 148.0 (8 C_{arom}), 125.8, 125.4 (4 C=), 142.4 , 142.3, 133.8, 132.9, 132.9, 132.7, 131.5, 129.2, 129.0, 113.4, 111.7, 111.6, 111.3 (24 CH_{arom}) ppm. $C_{18}H_{12}O_2Se_2$ (418.21): calcd. C 51.70, H 2.89; found: C 51.69, H 3.20.

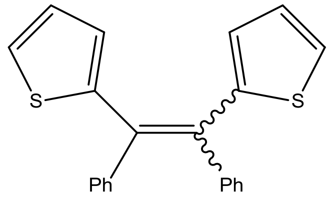

1,2-Diphenyl-1,2-bis(thiophen-2-yl)ethene (**9e**,[S3] mixture of *E*/*Z* isomers, ratio 3:2): Yield: 128 mg (75%); chromatografic purification (petroleum ether/CH_2Cl_2 8:2). Yellow crystals; m.p. 157–161 °C. ^1H NMR (600 MHz, $CDCl_3$): δ = 7.38 (br.s, 10 H_{arom}), 7.25 (dd, J = 4.8 Hz, 1.2 Hz, 2 H_{arom}), 7.10 (br.s, 10 H_{arom}), 7.05 (dd, J = 4.8 Hz, 1.2 Hz, 2 H_{arom}), 6.88 (dd, J = 4.8 Hz, 3.6 Hz, 2 H_{arom}), 6.79 (dd, J = 3.6 Hz, 1.2 Hz, 2 H_{arom}), 6.70 (dd, J = 4.8 Hz, 3.6 Hz, 2 H_{arom}), 6.37 (dd, J = 3.6 Hz, 1.2 Hz, 2 H_{arom}) ppm. ^{13}C NMR (150 MHz, $CDCl_3$): δ = 145.9, 145.8, 142.7, 142.3, 134.7, 133.1 (8 C_{arom}, 4 C=), 131.0, 130.9, 129.7, 129.4, 128.8, 128.0, 127.6, 126.9, 126.8, 126.7, 126.5, 125.8 (32 CH_{arom}) ppm.

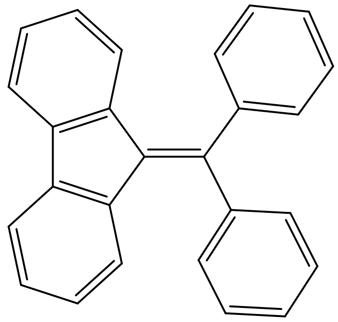

9-(Diphenylmethylene)-9H-fluorene (**9g**):[S4] Yield: 119 mg (72%); chromatografic purification (petroleum ether/ethyl acetate 9:1). Pale yellow solid; m.p. 226–228 °C (ref.[S4]: 226−228 °C). ^{1}H NMR (600 MHz, $CDCl_3$): δ = 7.71 (d, J = 7.8 Hz, 2 H_{arom}), 7.38–7.46 (m, 10 H_{arom}), 7.23–7.26 (m, 2 H_{arom}), 6.92–6.96 (m, 2 H_{arom}), 6.64 (d, J = 7.8 Hz, 2 H_{arom}) ppm. ^{13}C NMR (150 MHz, $CDCl_3$): δ = 145.5, 143.0, 138.7, 134.2, 127.6 (5 signals for 8 C_{arom}), 140.5, 129.6, 128.8, 128.2, 126.4, 124.9, 119.2 (7 signals for 18 CH_{arom}) ppm.

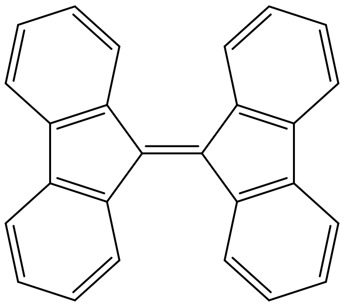

9,9'-Bis(fluorenylidene) (**9h**):[S5] Yield: 115 mg (70%); chromatografic purification (petroleum ether/ethyl acetate 8:2). Orange solid; m.p. 174–177 °C (ref.[S5]: 175−178 °C). ^{1}H NMR (600 MHz, $CDCl_3$): δ = 8.40 (d, J = 7.8 Hz, 4 H_{arom}), 7.72 (d, J = 7.8 Hz, 4 H_{arom}), 7.32–7.36 (m, 4 H_{arom}), 7.20–7.24 (m, 4 H_{arom}) ppm. ^{13}C NMR (150 MHz, $CDCl_3$): δ = 141.3, 141.0, 138.3 (3 signals for 10 C_{arom}), 129.1, 126.8, 126.7, 119.9 (4 signals for 16 CH_{arom}) ppm.

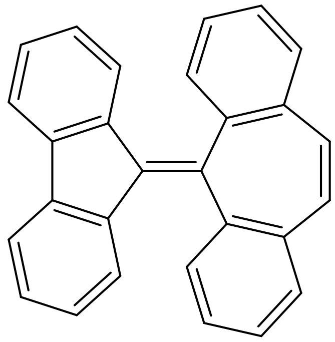

5-(9H-Fluoren-9-ylidene)-5H-dibenzo[a,d][7]annulene (tetrabenzosesquifulvalene) (**9j**)[S6]: Yield: 117 mg (66 %); Pale yellow solid; m.p. 300–302 °C (ref.[S6]: 298−300 °C). ^{1}H NMR (600 MHz, $CDCl_3$): δ = 7.65 (d, J = 7.2 Hz, 2 H_{arom}), 7.60 (dd, J = 1.2 Hz, J= 7.2 Hz, 2 H_{arom}), 7.54 (dd, J = 1.2 Hz, J= 7.2 Hz, 2 H_{arom}), 7.42–7.50 (m, 4 H_{arom}), 7.23 (dt, J = 0.6 Hz, J= 7.8 Hz, 2 H_{arom}), 7.05 (s, 2 H_{arom}), 6.91 (dt, J = 0.6 Hz, J=7.8 Hz, 2 H_{arom}), 6.47 (d, J = 7.8 Hz, 2 H_{arom}) ppm. ^{13}C NMR (150 MHz, $CDCl_3$): δ =

141.2, 140.6, 138.0, 137.9, 133.6, 132.5 (6 signals for 10 C_{arom}),130.8, 128.6, 128.4, 127.8, 127.4, 127.0, 126.4, 125.1, 119.2 (9 signals for 18 CH_{arom}), ppm.

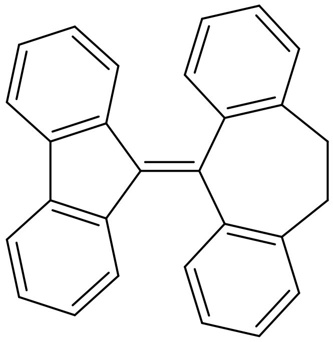

5-(9H-Fluoren-9-ylidene)-10,11-dihydro-5H-dibenzo[a,d][7]annulene (**9k**)[S7]: Yield: 220 mg (62 %); Pale yellow solid; m.p. 284−286 °C (ref.[S7]: 296 °C). ^{1}H NMR (600 MHz, $CDCl_3$): δ = 7.72 (d, J = 7.8 Hz, 2 H_{arom}), 7.44 (d, J= 7.2 Hz, 2 H_{arom}), 7.26-7.34 (m, 6 H_{arom}), 7.21–7.25 (m, 2 H_{arom}), 6.95–7.00 (m, 2 H_{arom}), 6.88 (dd, J = 0.6 Hz, J=7.8 Hz, 2 H_{arom}), 3.44–3.53 (m, 2 H_{arom}), 2.85–2.92 (m, 2 H_{arom}) ppm. ^{13}C NMR (150 MHz, $CDCl_3$): δ = 144.6, 141.6, 140.5, 138.2, 136.7, 132.2 (6 signals for 10 C_{arom}), 130.1, 128.1, 127.7, 127.6, 126.4, 126.1, 125.3, 119.2 (8 signals for 16 CH_{arom}), 31.9 (1 signal for 2 CH_2) ppm.

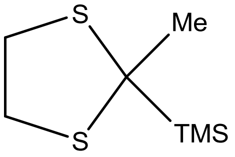

2-Methyl-2-(trimethylsilyl)-1,3-dithiolane (**13a**): Yield: 130 mg (68%); colorless solid, m.p. 46–47 °C. IR (KBr): ν = 2949 (s), 2927 (m), 2855 (w), 1442 (m), 1369 (w), 1245 (s), 1097 (w), 1059 (w), 1006 (w), 840 (s), 755 (m), 701 (m), 620 (w) cm^{-1}. ^{1}H NMR (600 MHz, $CDCl_3$): δ = 3.31–3.36 (m, 2H), 3.14–3.19 (m, 2H), 1.61 (s, 3H), 0.17 (s, 9H) ppm. $C_7H_{16}S_2Si$ (192.42): calcd. C 43.69, H 8.38, S 33.33 ; found: C 43.63, H 8.31, S 33.44.

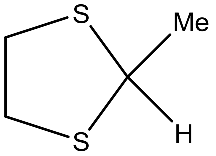

2-Methyl-1,3-dithiolane (**15a**): Yield: 35 mg (58 %); colorless oil (ref.[S8], colorless oil, b.p. 76 °C/23 Torr). ^{1}H NMR (600 MHz, $CDCl_3$): δ = 4.63 (q, J = 6.6 Hz, 1 H), 3.21–

3.36 (m, 4 H), 1.63 (d, J = 6.6 Hz, 3 H) ppm. ^{13}C NMR (150 MHz, $CDCl_3$): δ = 48.2 (CH), 39.1 (CH_2CH_2), 24.7 (CH_3) ppm.

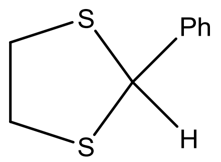

2-Phenyl-1,3-dithiolane (**15b**): Yield: 68 mg (74 %) (colorless oil, chromatographic purification: petroleum ether/CH_2Cl_2 9:1) (ref.[S9, S10], colorless oil). ^{1}H NMR (600 MHz, $CDCl_3$): δ = 7.44–7.45 (m, 2H), 7.17–7.24 (m, 3H), 5.57 (s, 1H), 3.25–3.45 (m, 4 H) ppm.

References

[S1] S. Rele, S. Talukdar, A. Banerji, S. Chattopadhyay, *J. Org. Chem.* **2001**, *66*, 2990−2994.
doi: 10.1021/jo001586a

[S2] T. Suzuki, H. Shiohara, M. Monobe, T. Sakimura, S. Tanaka, Y. Yamashita, T. Miyashi, *Angew. Chem. Int. Ed.* **1992**, *31*, 455–458.
doi: 10.1002/anie.199204551

[S3] E. Fischer, J. Larsen, J. B. Christensen, M. Fourmigué, H. G. Madsen, N. Harrit, *J. Org. Chem*. **1996**, *61*, 6997–7005.
doi: 10.1021/jo960022x

[S4] A. Schönberg, K.-H. Brosowski, E. Singer, *Chem. Ber*. **1962**, *95*, 2144–2154.
doi: 10.1002/cber.19620950907

[S5] H.-J. Lubberger, E. Mueller, J. Hofmann, H. Fischer, J. C. Jochims, *Chem. Ber.* **1991**, *124*, 2537−2544.
doi: 10.1002/cber.19911241124

[S6] L. Salisbury, *J. Org. Chem.* **1970**, *35*, 4258−4259.
doi: 10.1021/jo00837a636

[S7] M. L. T. M. B. Franco, B. J. Herold, J. C. Evans, Ch. C. Rowlands, J. Chem. *Soc.* Perkin Trans. II **1988**, 443−450.
doi: 10.1039/p29880000443

[S8] K.-D. Asmus, D. Bahnemann, Ch.-H. Fischer, D. Veltwisch, *J. Am. Chem. Soc.* **1979**, *101*, 5322−5329.

doi: 10.1021/ja00512a035

[S9] H. Tani, K. Masumoto, T. Inamasu, *Tetrahedron Lett*. **1991**, *32*, 2039−2042. doi: 10.1016/S0040-4039(00)78902-7

[S10] M. H. Ali, M. G. Gomes, *Synthesis* **2005**, 1326−1332. doi: 10.1055/s-2005-865303

2. Copies of ^{1}H and ^{13}C NMR spectra for compounds **9**, **13**, and **15**

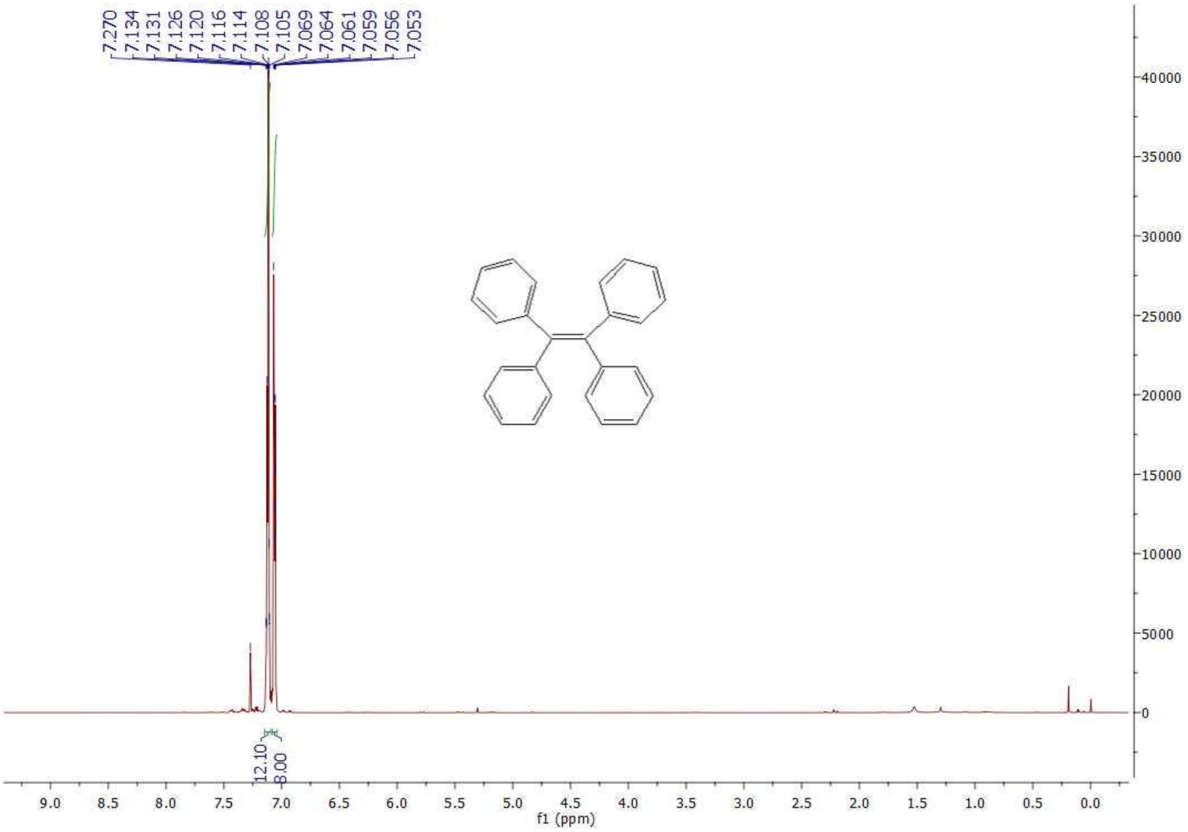

Figure S1. The ^{1}H NMR spectrum of compound **9a.**

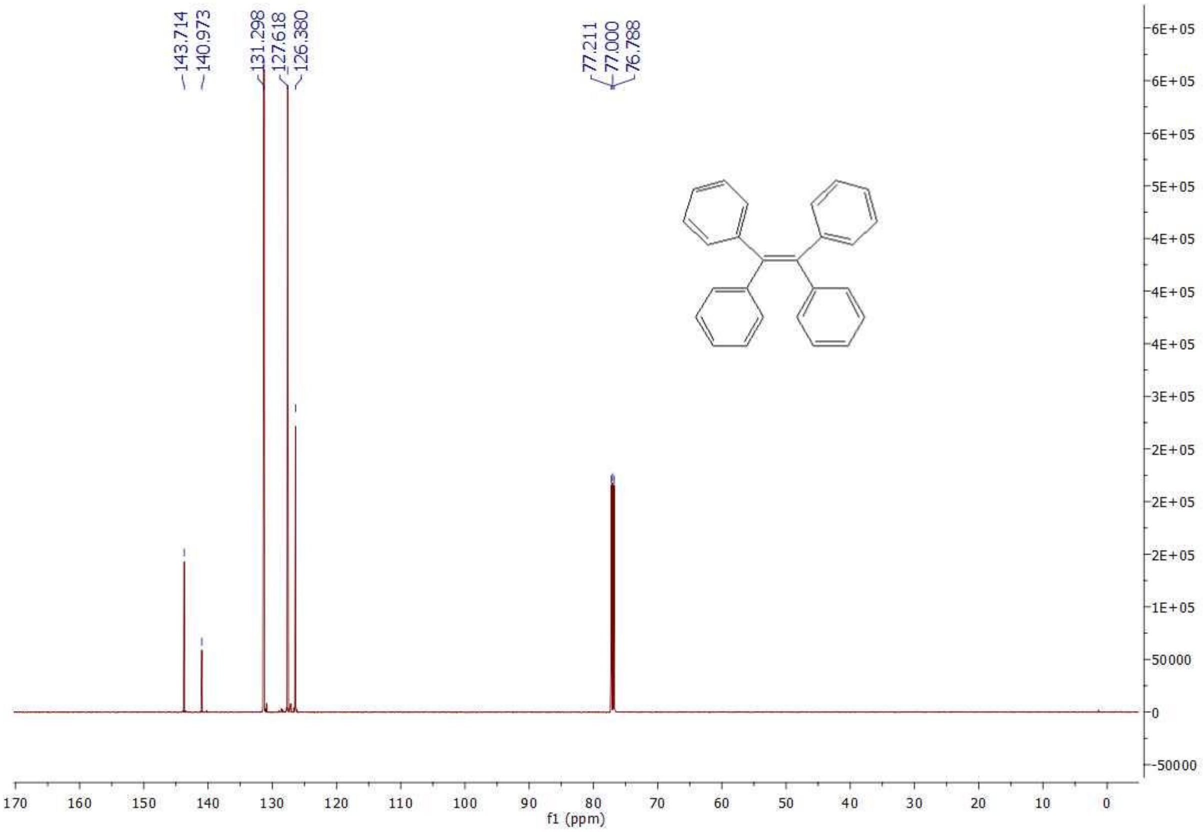

Figure S2. The ^{13}C NMR spectrum of compound **9a**.

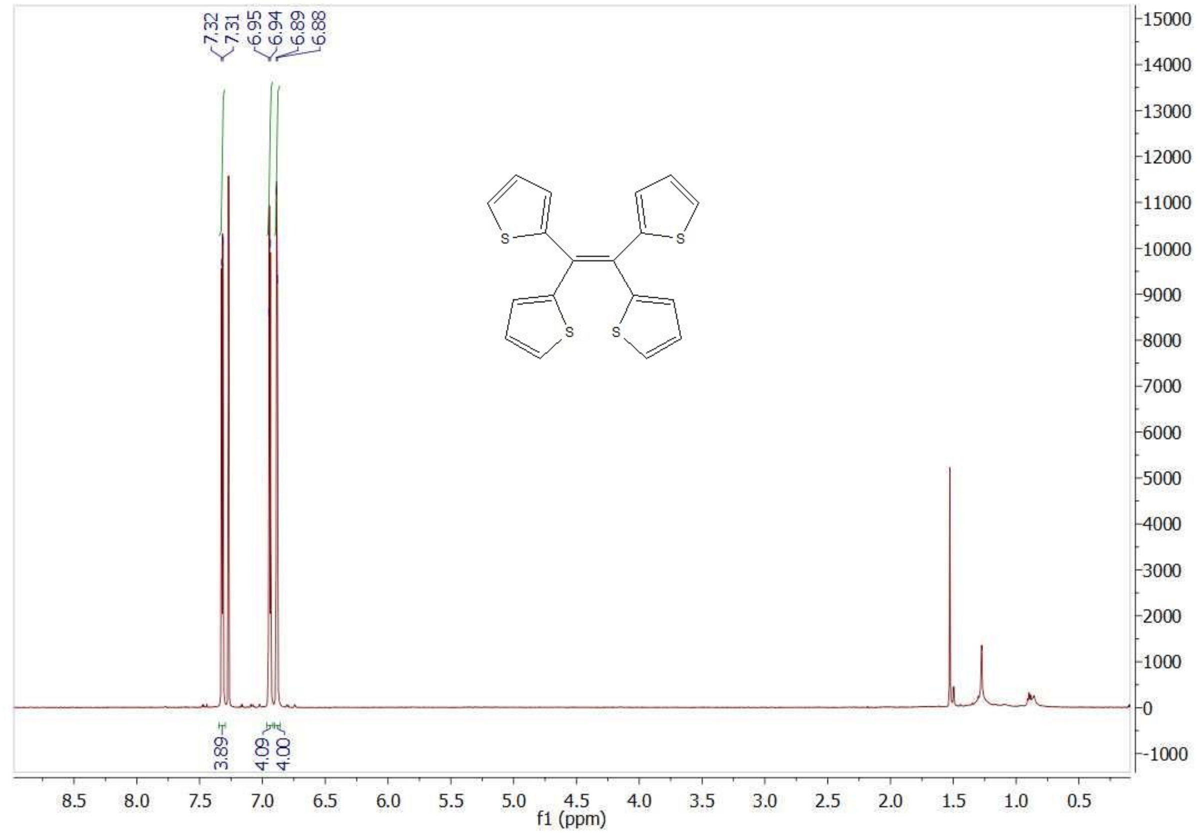

Figure S3. The ^{1}H NMR spectrum of compound **9b.**

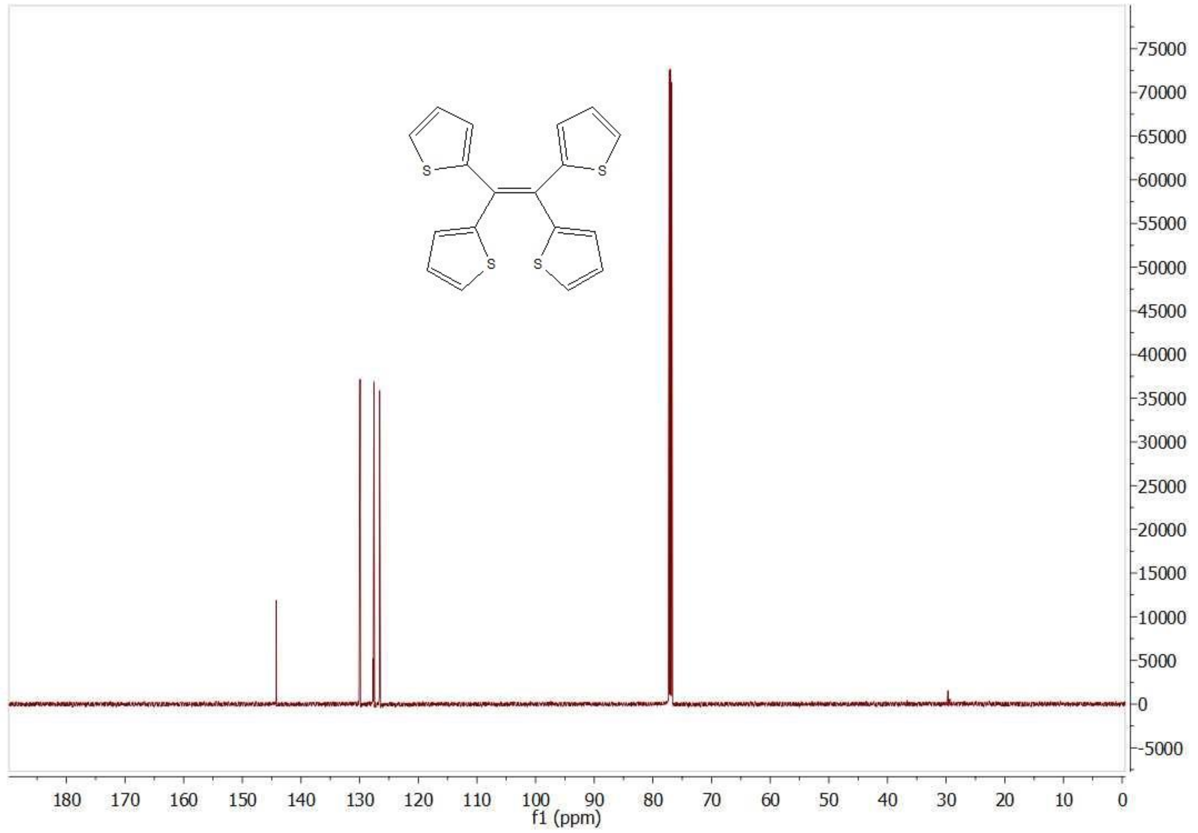

Figure S4. The ^{13}C NMR spectrum of compound **9b**.

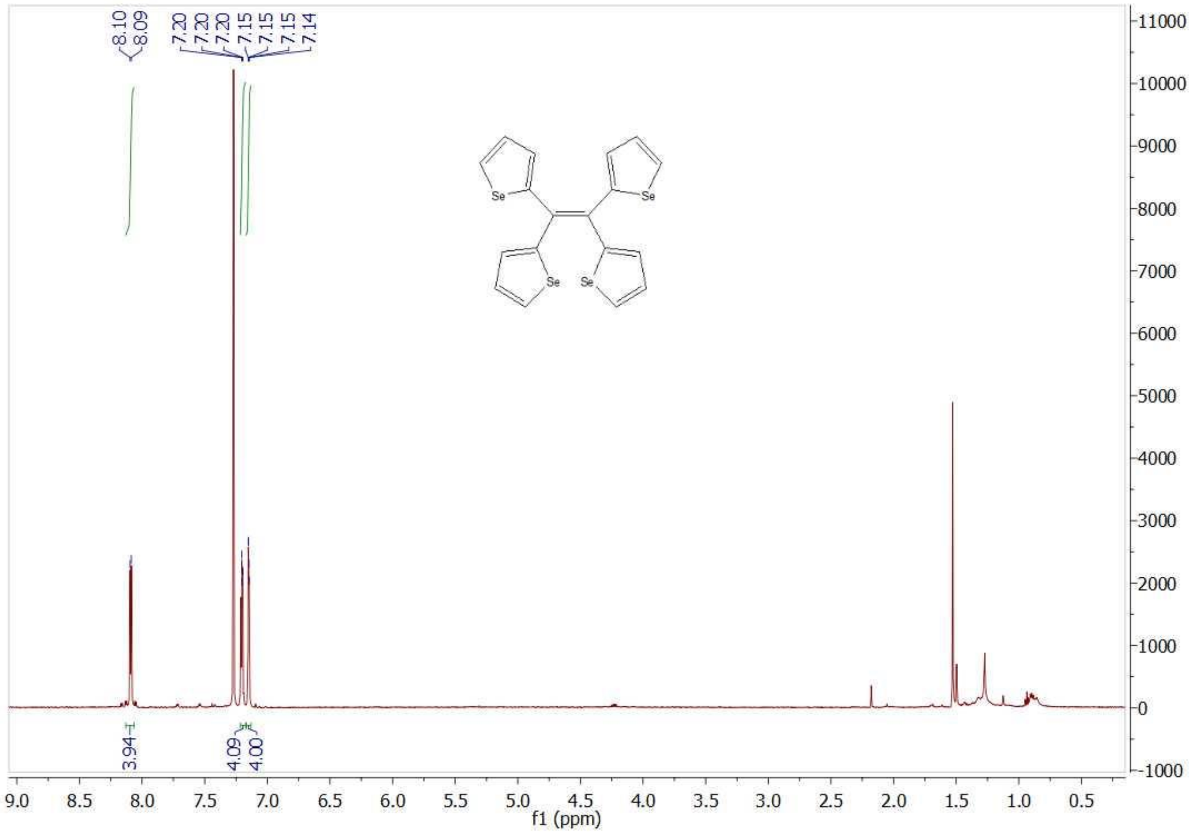

Figure S5. The ^{1}H NMR spectrum of compound **9c.**

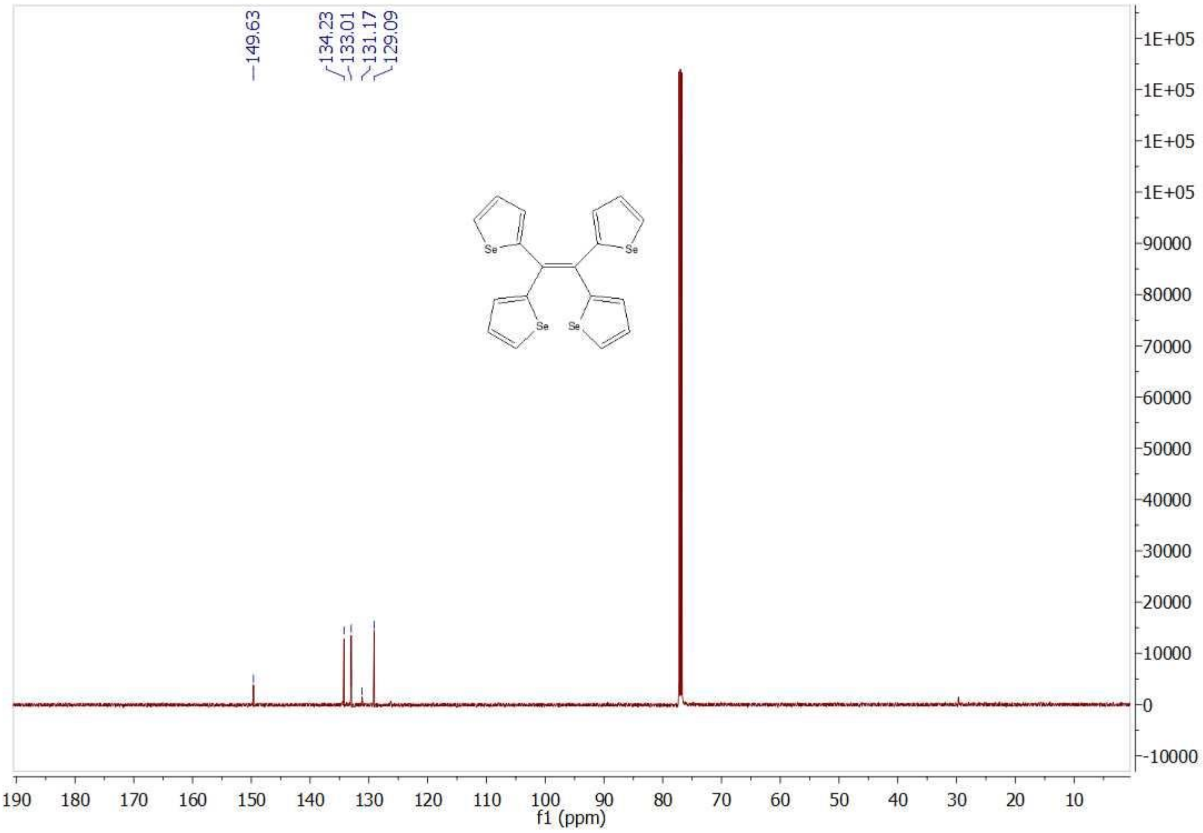

Figure S6. The ^{13}C NMR spectrum of compound **9c**.

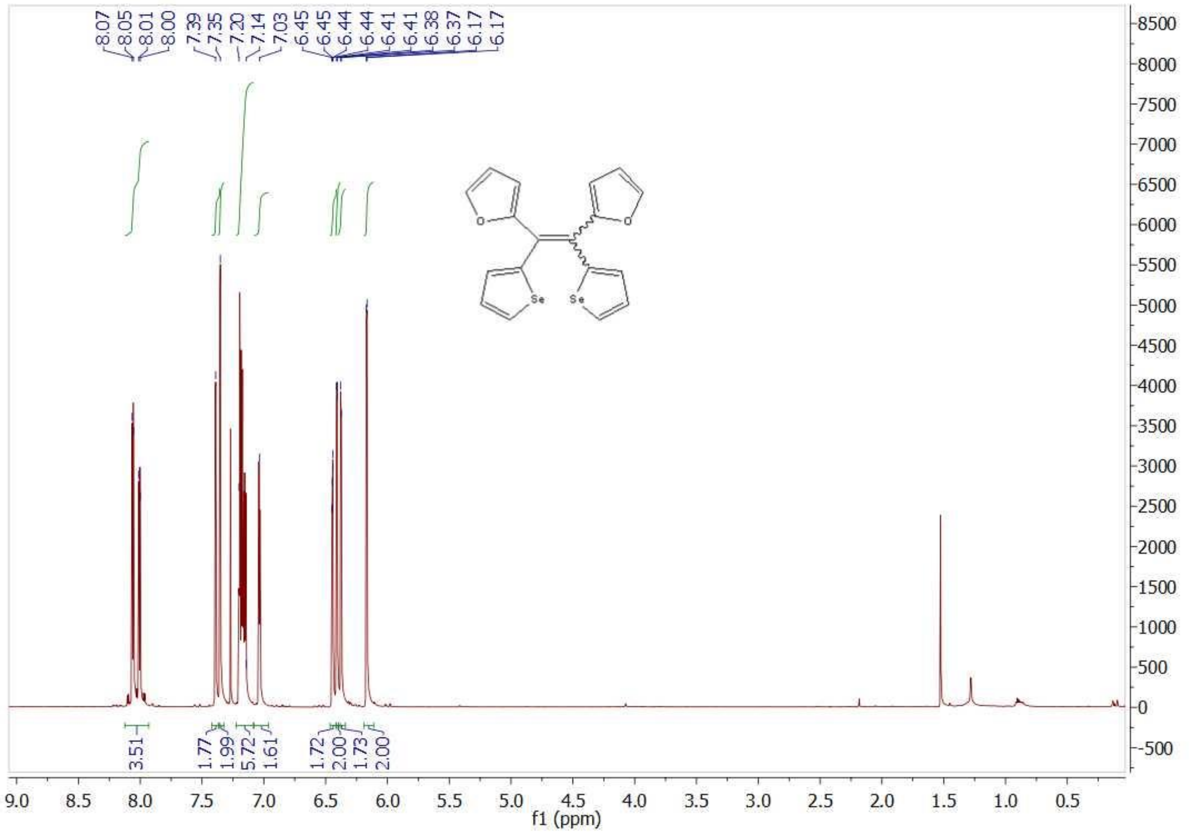

Figure S7. The ^{1}H NMR spectrum of compound **9d.**

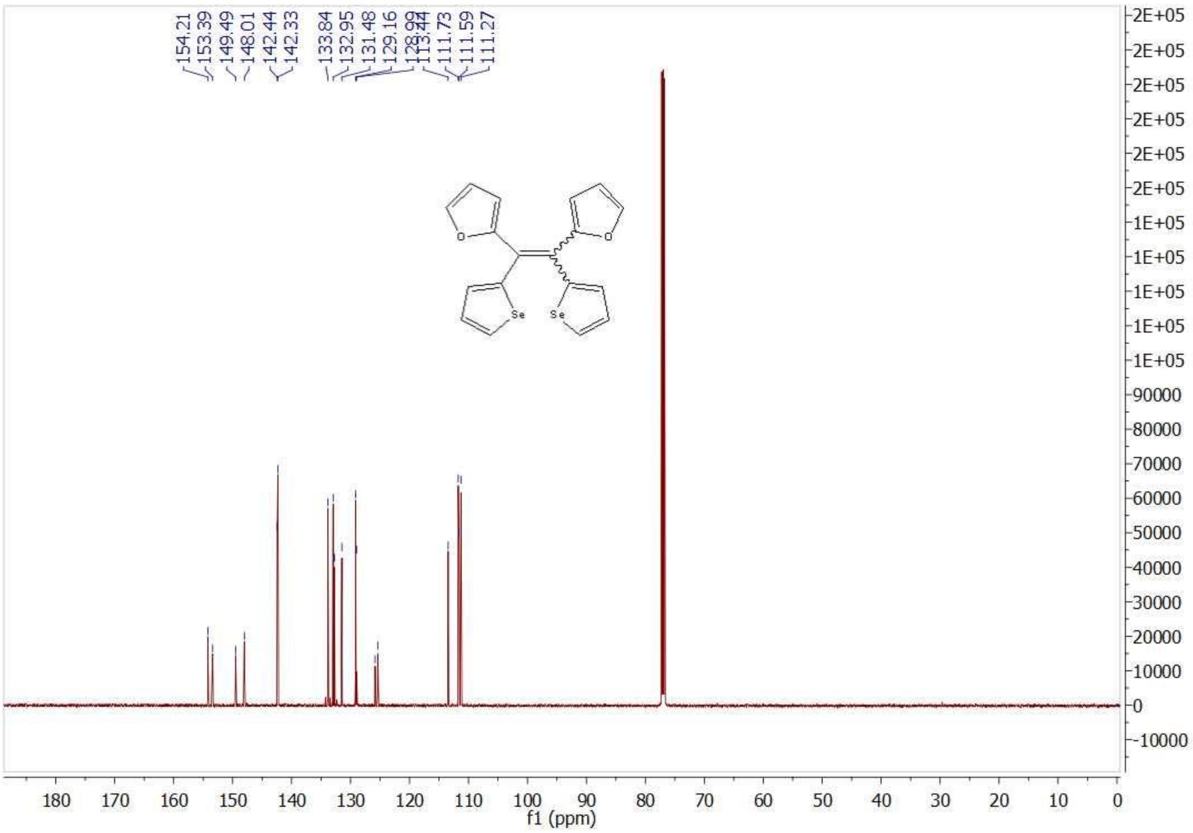

Figure S8. The ^{13}C NMR spectrum of compound **9d**.

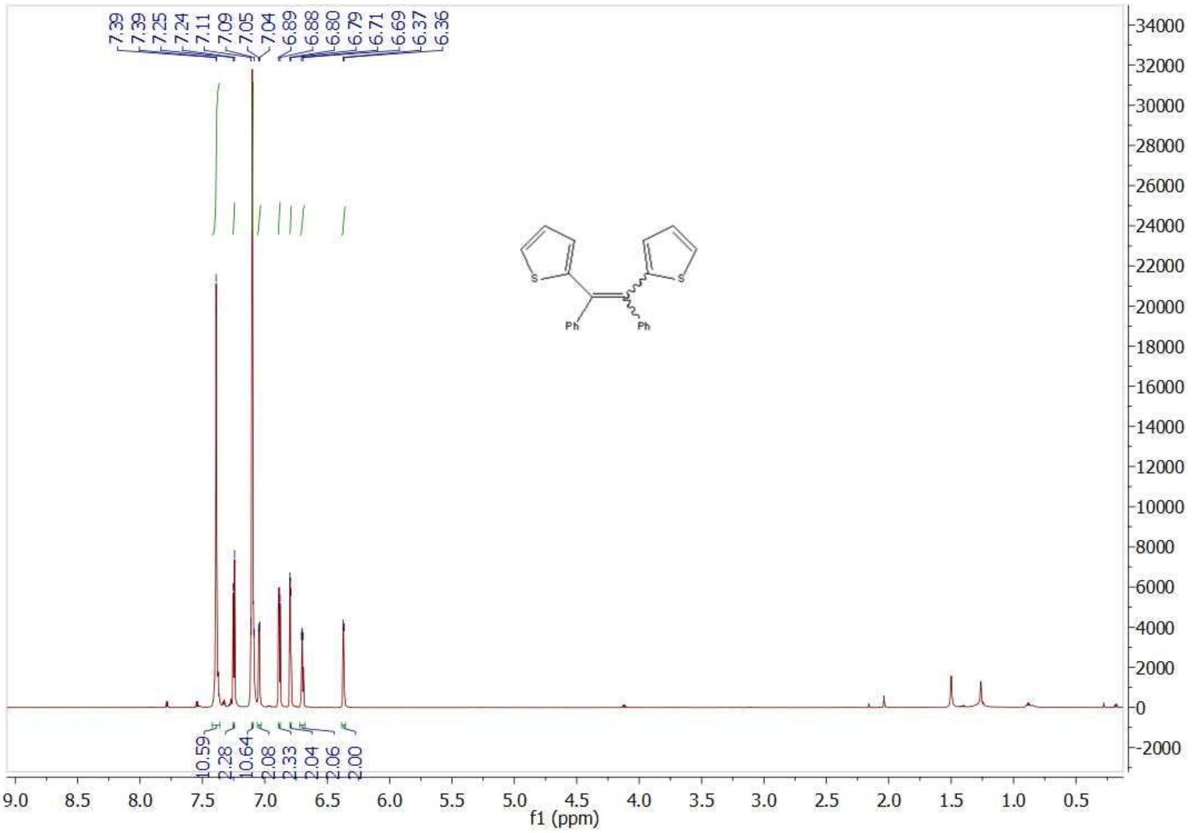

Figure S9. The ^{1}H NMR spectrum of compound **9e.**

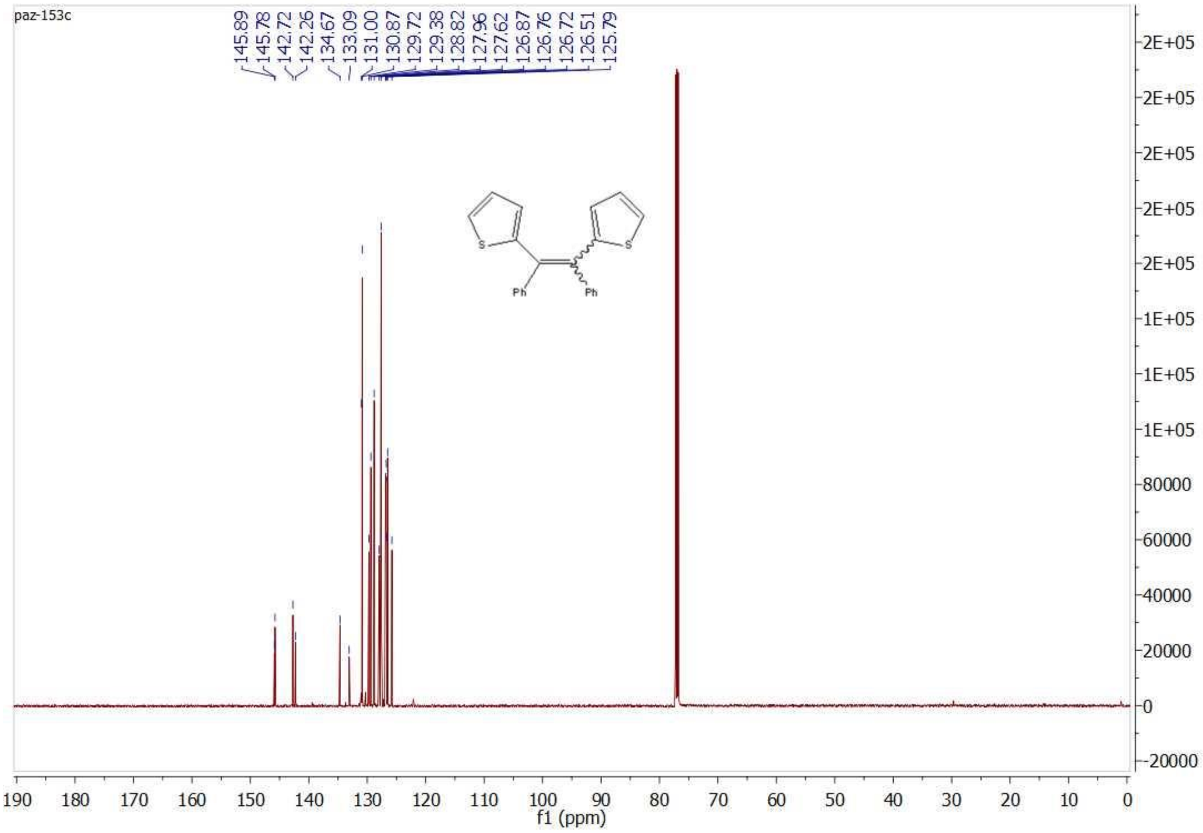

Figure S10. The ^{13}C NMR spectrum of compound **9e**.

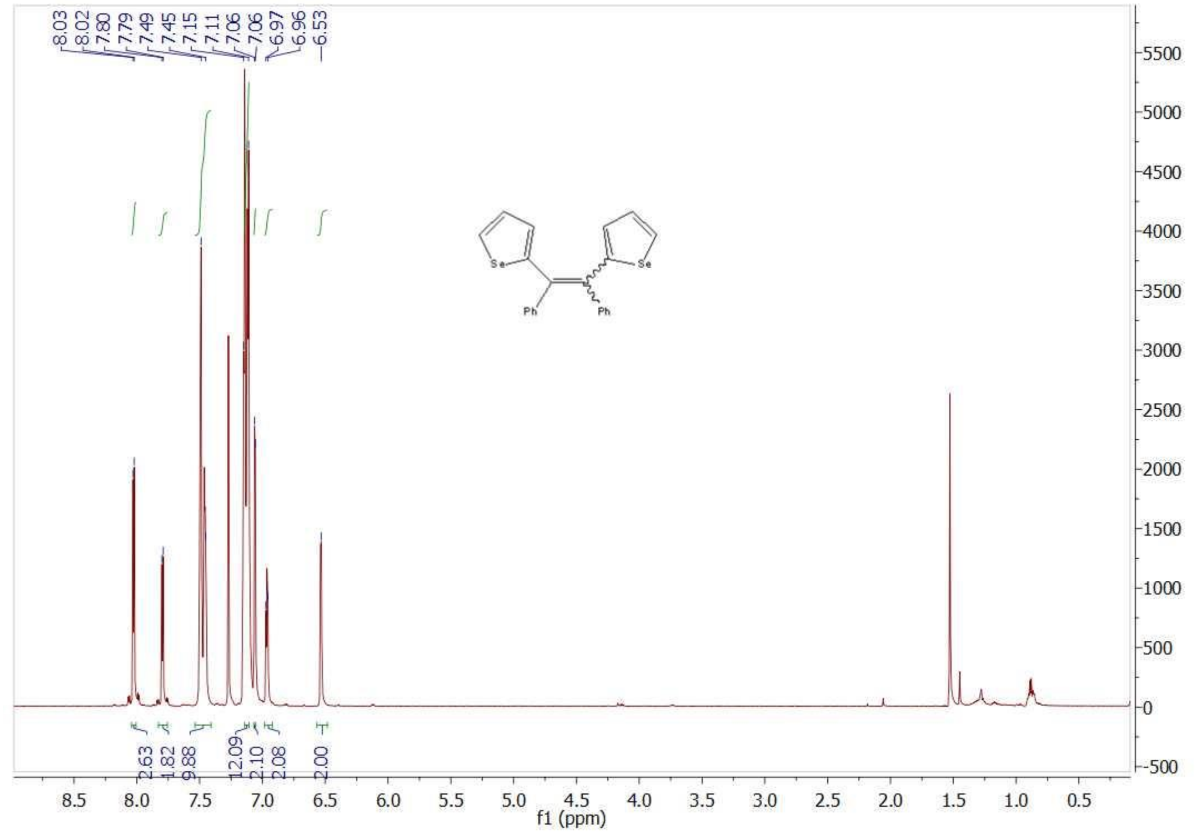

Figure S11. The ^{1}H NMR spectrum of compound **9f.**

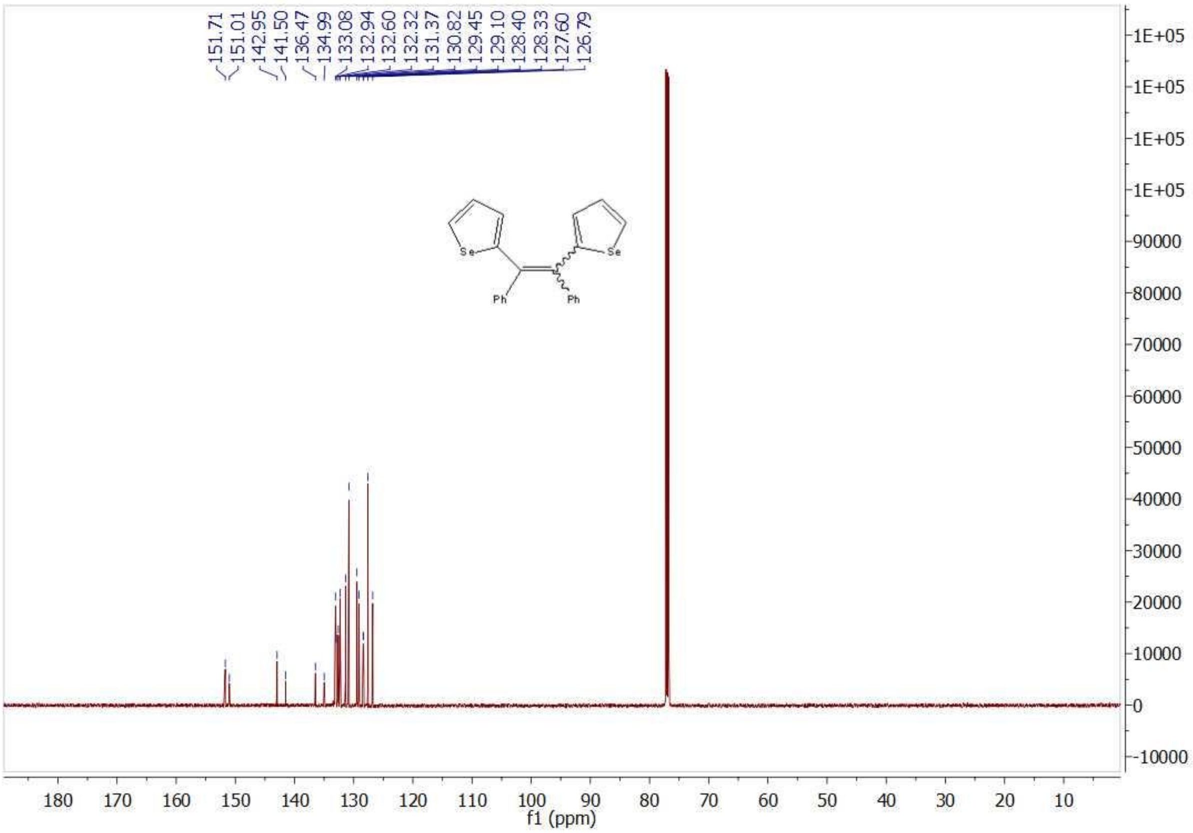

Figure S12. The ^{13}C NMR spectrum of compound **9f**.

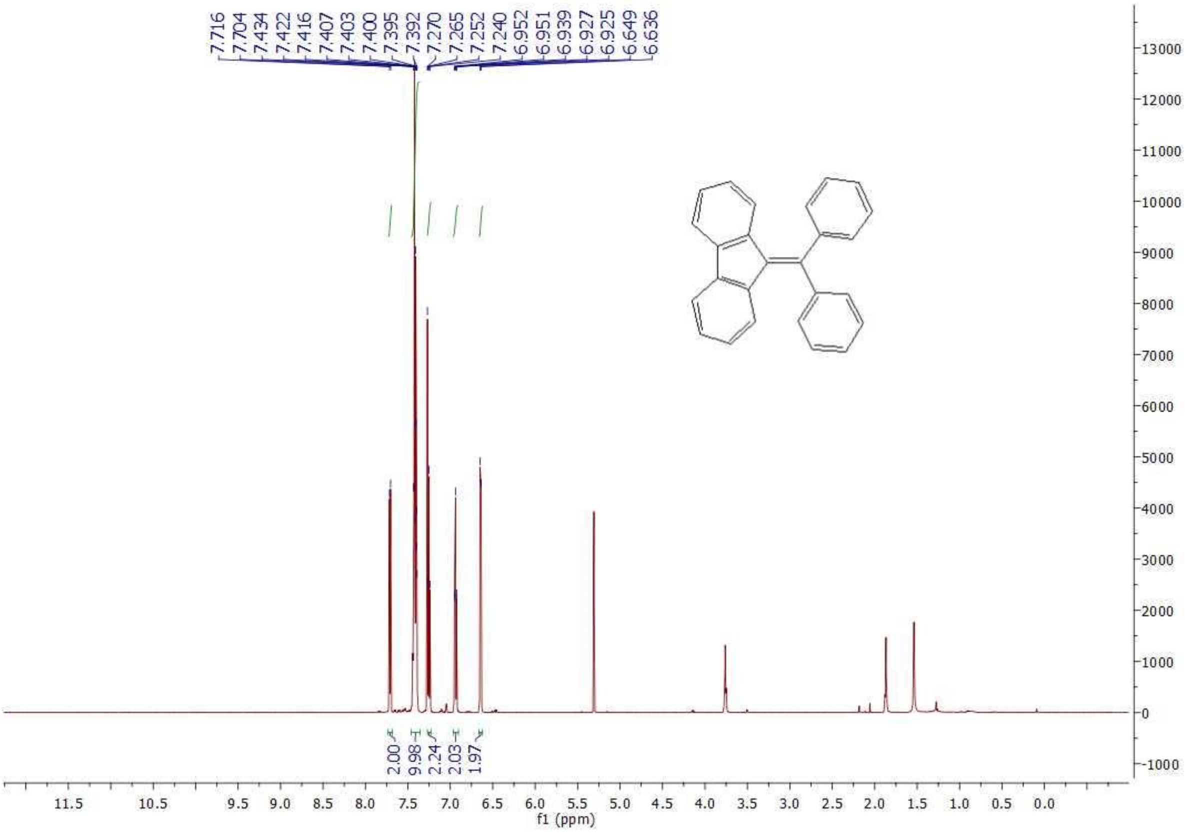

Figure S13. The ^{1}H NMR spectrum of compound **9g.**

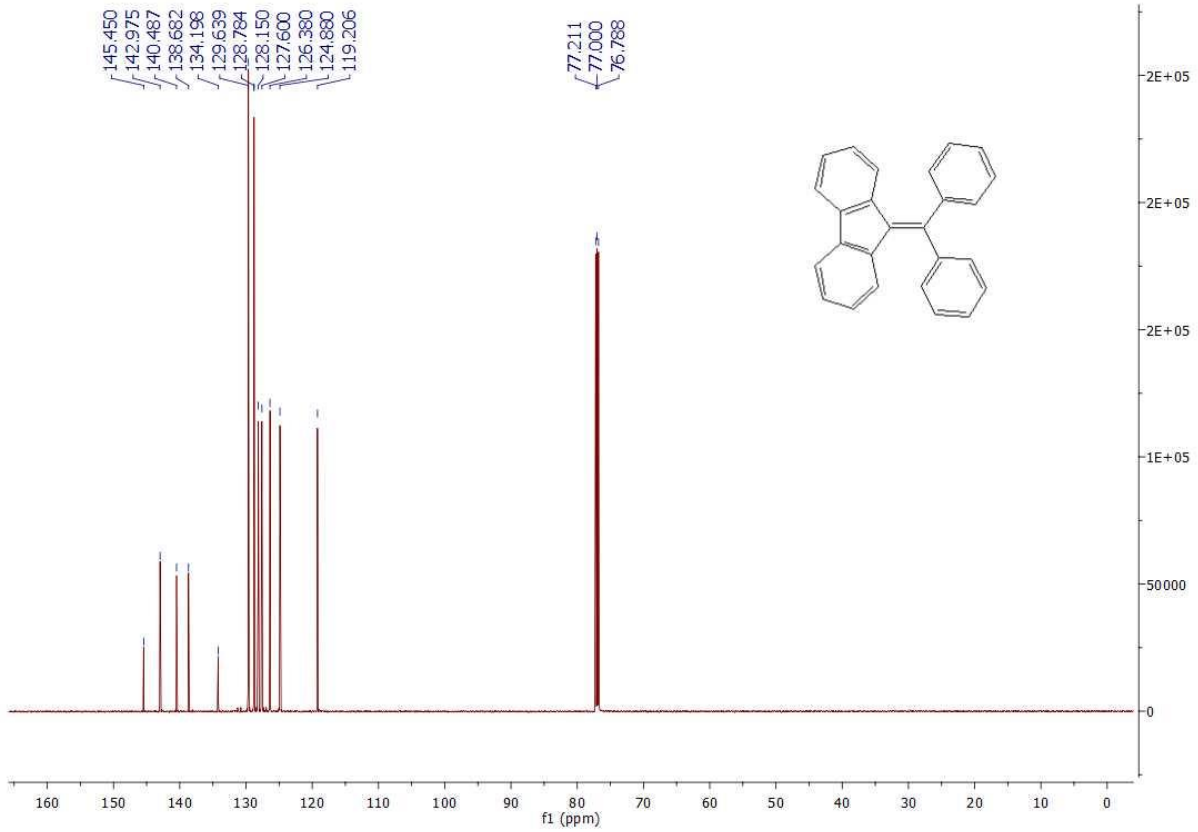

Figure S14. The ^{13}C NMR spectrum of compound **9g**.

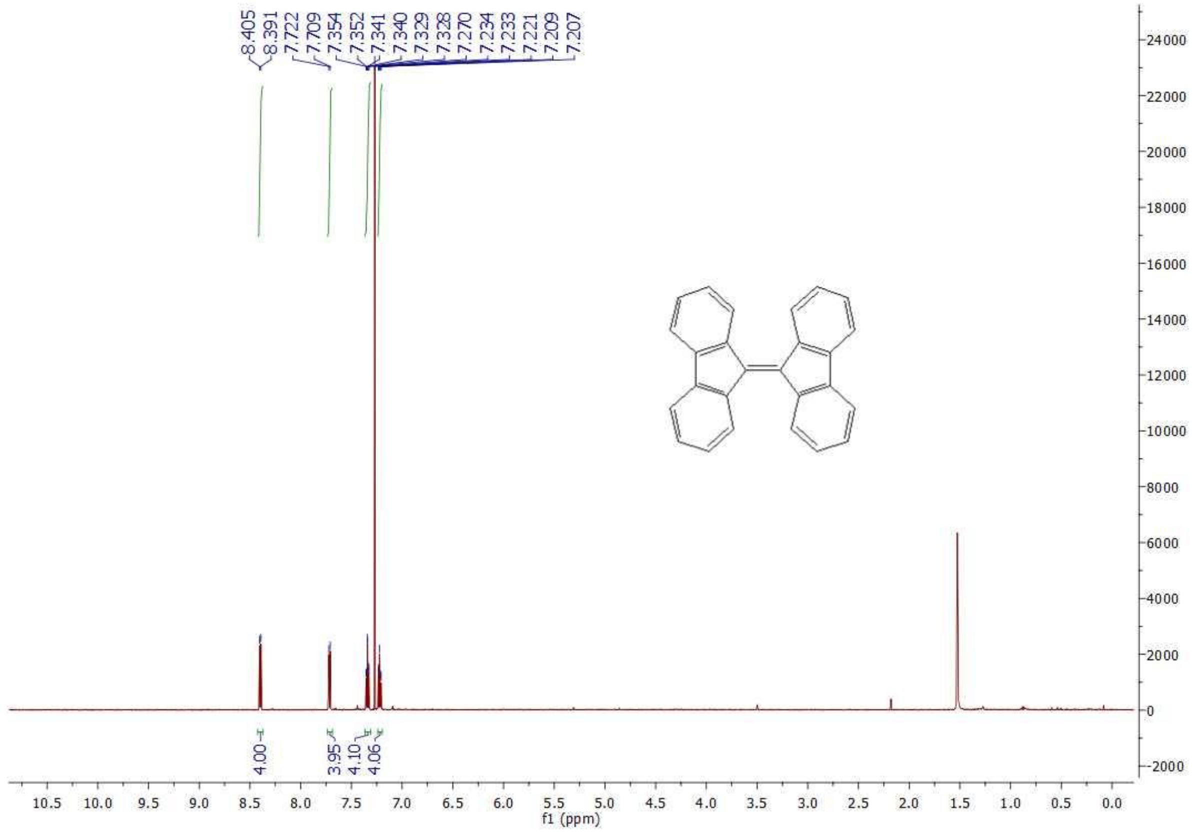

Figure S15. The ^{1}H NMR spectrum of compound **9h.**

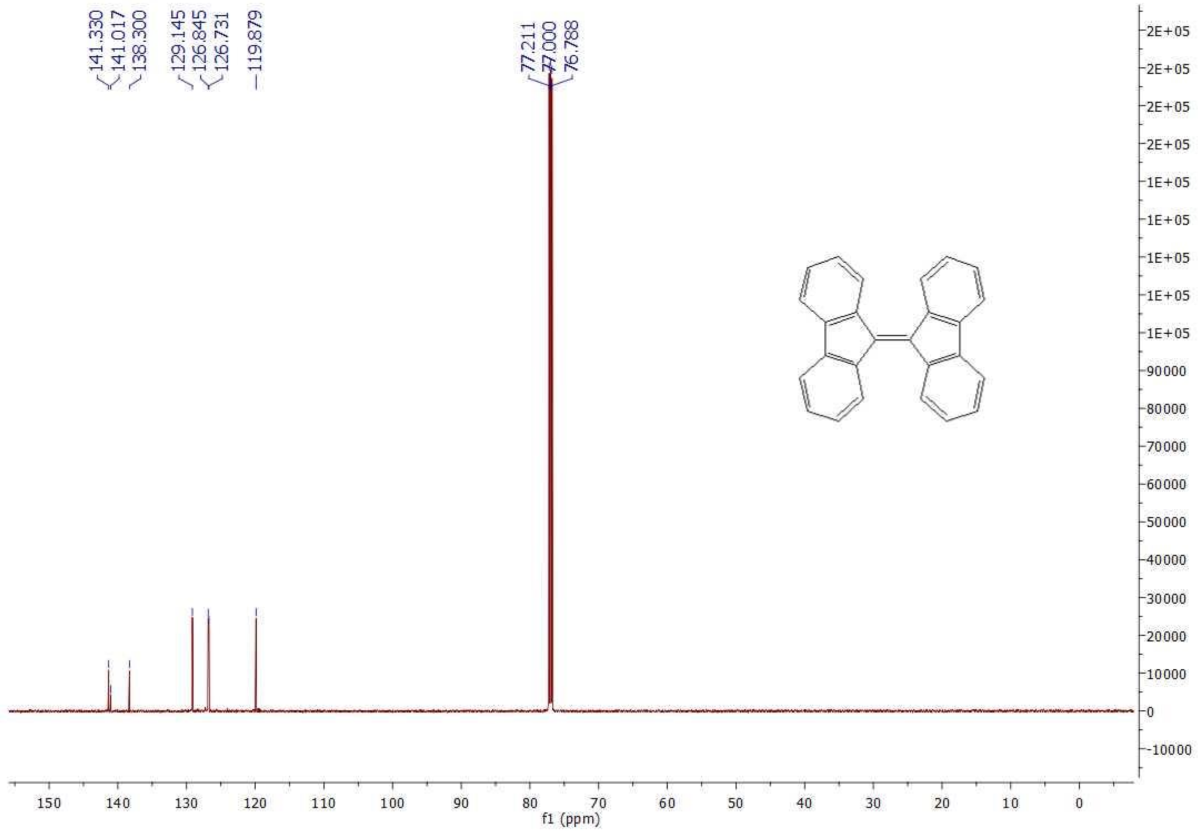

Figure S16. The ^{13}C NMR spectrum of compound **9h**.

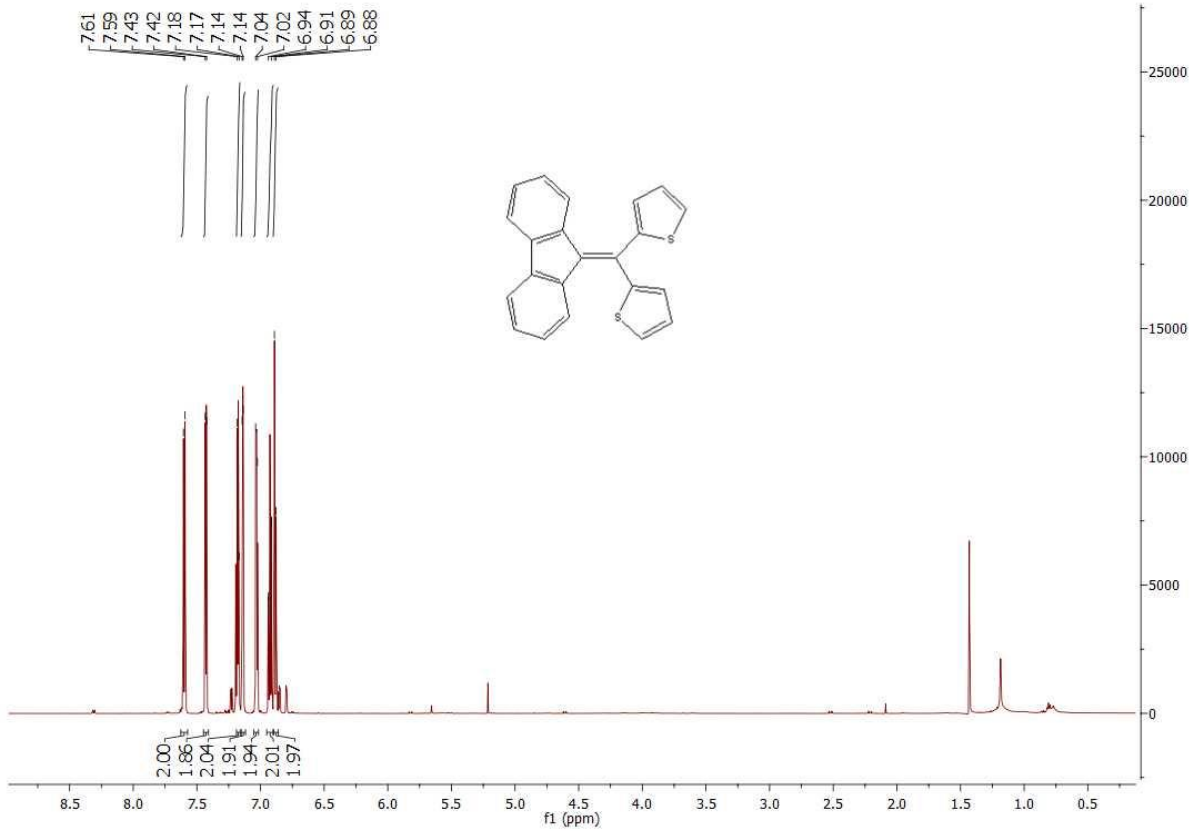

Figure S17. The ^{1}H NMR spectrum of compound **9i.**

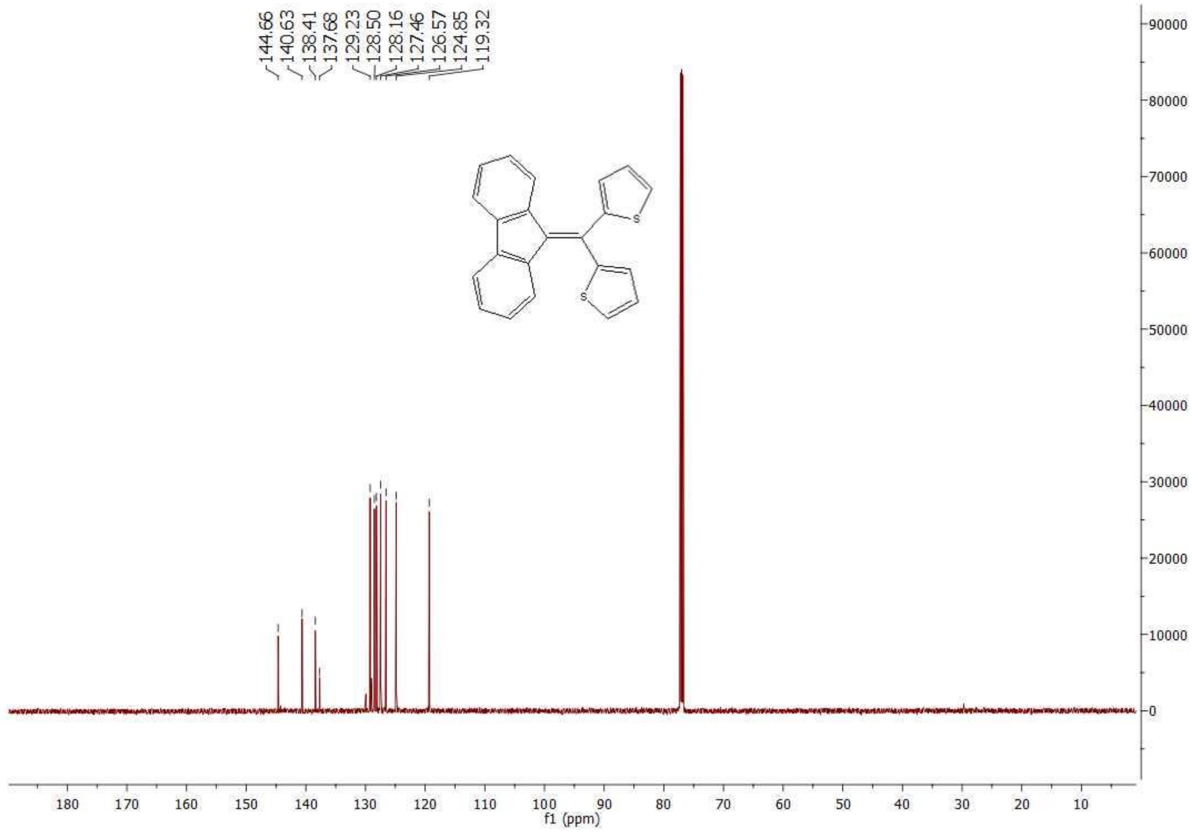

Figure S18. The ^{13}C NMR spectrum of compound **9i**.

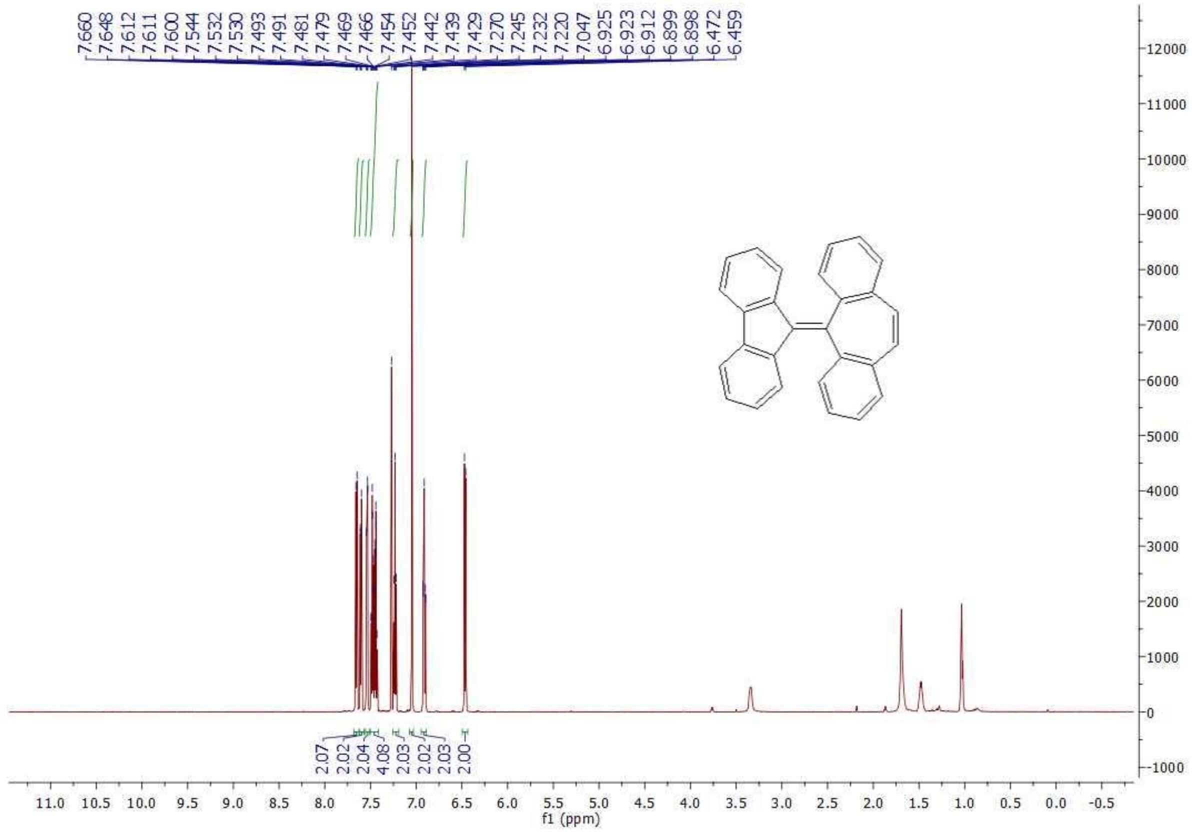

Figure S19. The ^{1}H NMR spectrum of compound **9j.**

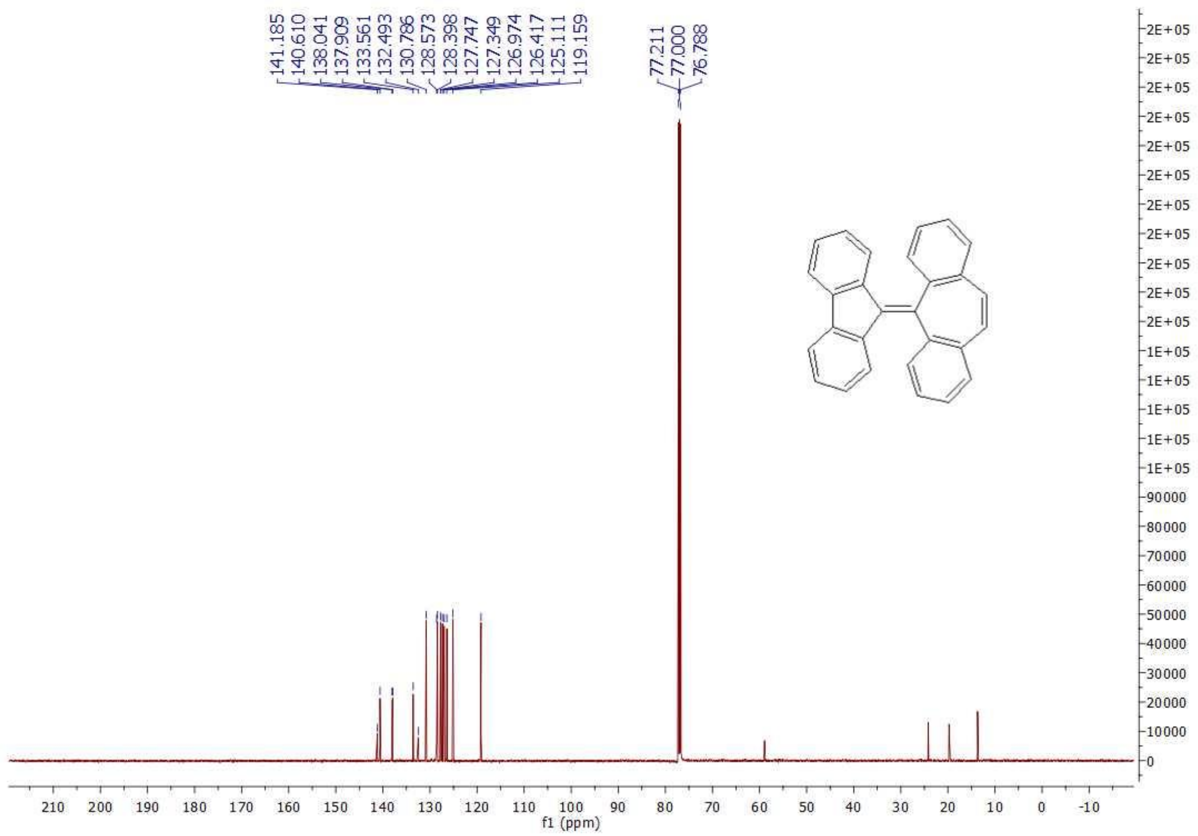

Figure S20. The ^{13}C NMR spectrum of compound **9j**.

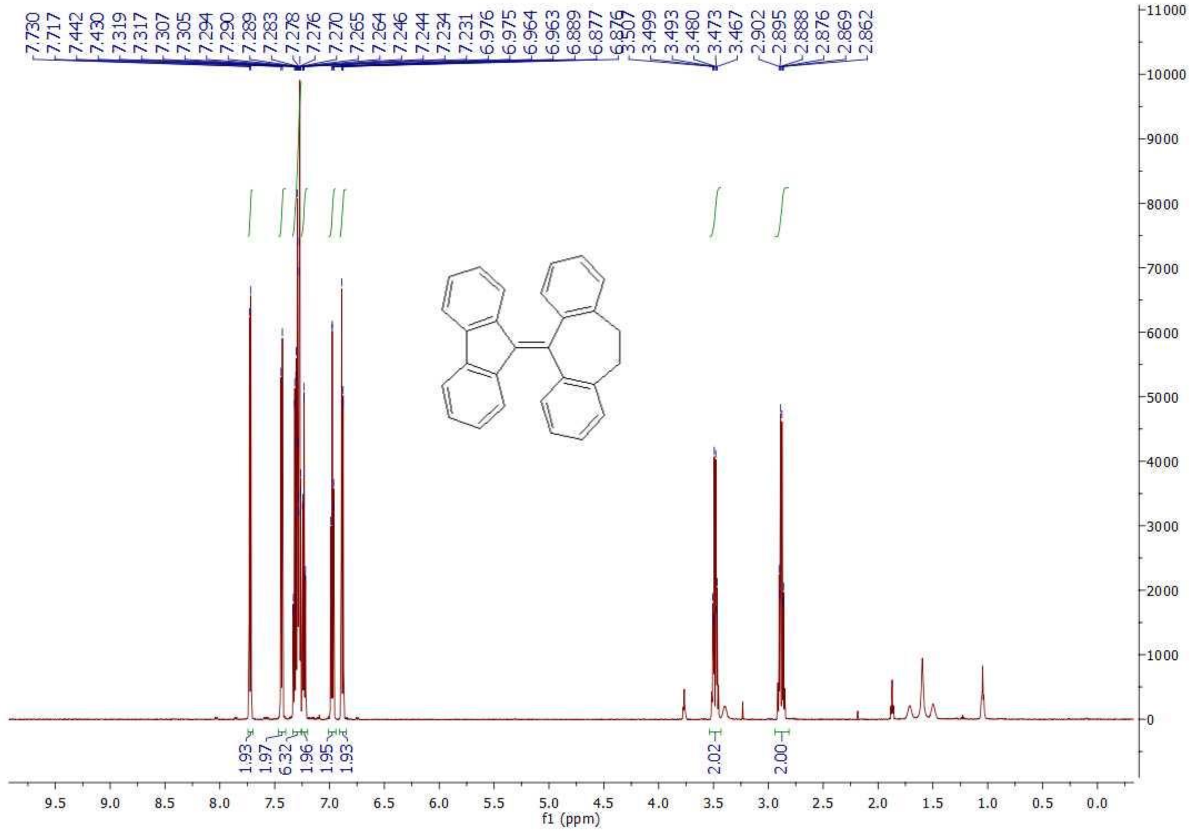

Figure S21. The ^{1}H NMR spectrum of compound **9k.**

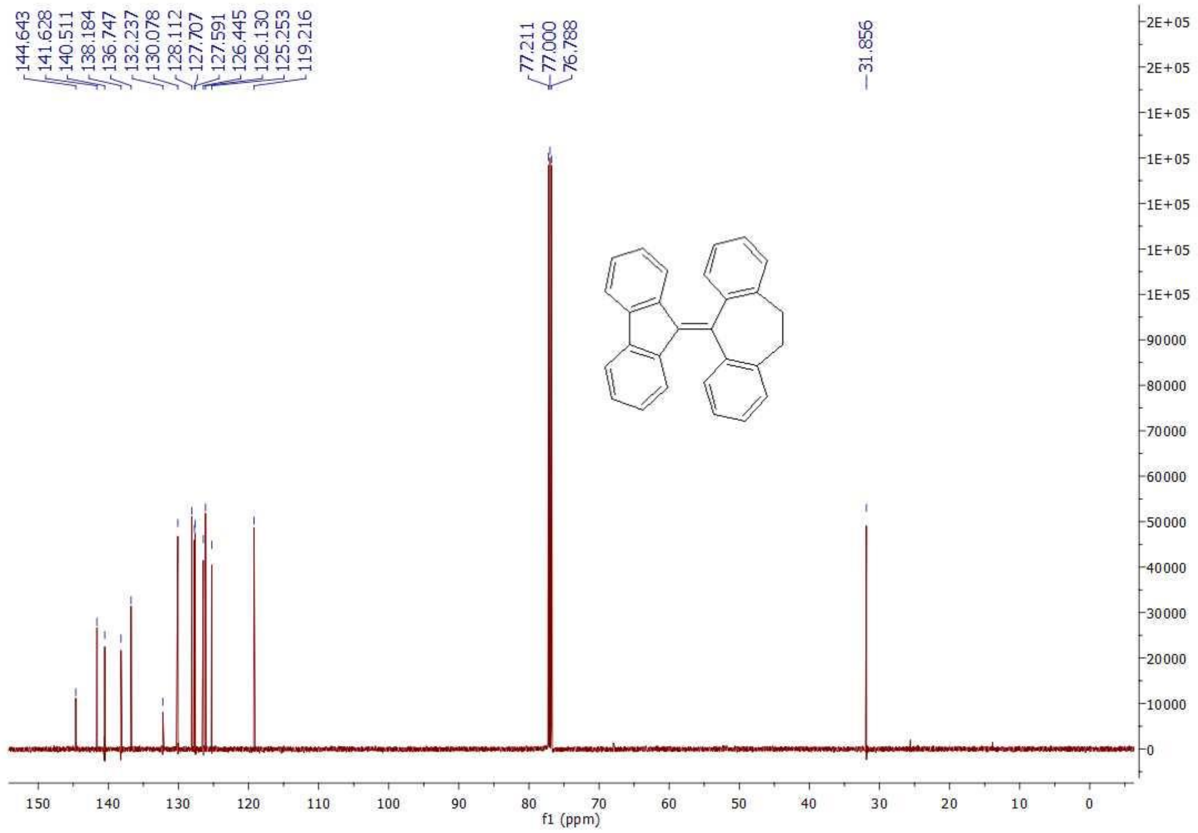

Figure S22. The ^{13}C NMR spectrum of compound **9k**.

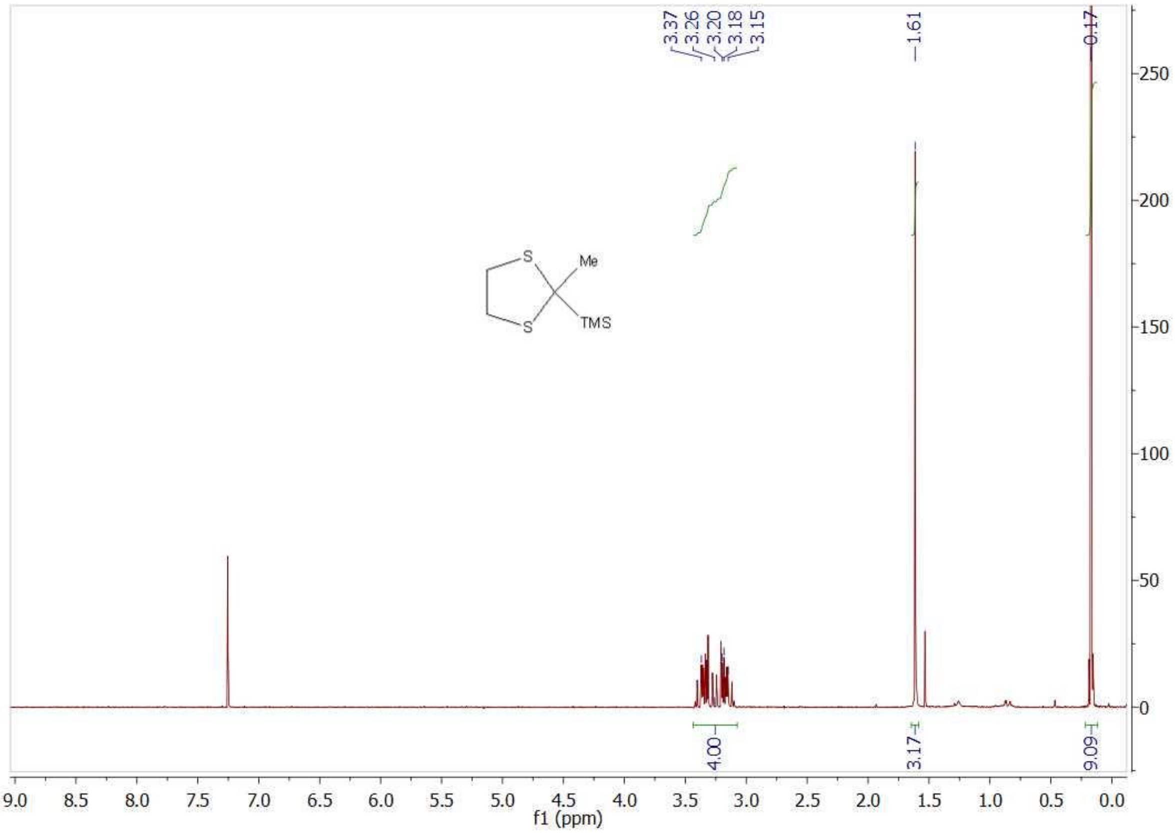

Figure S23. The ^{1}H NMR spectrum of compound **13a.**

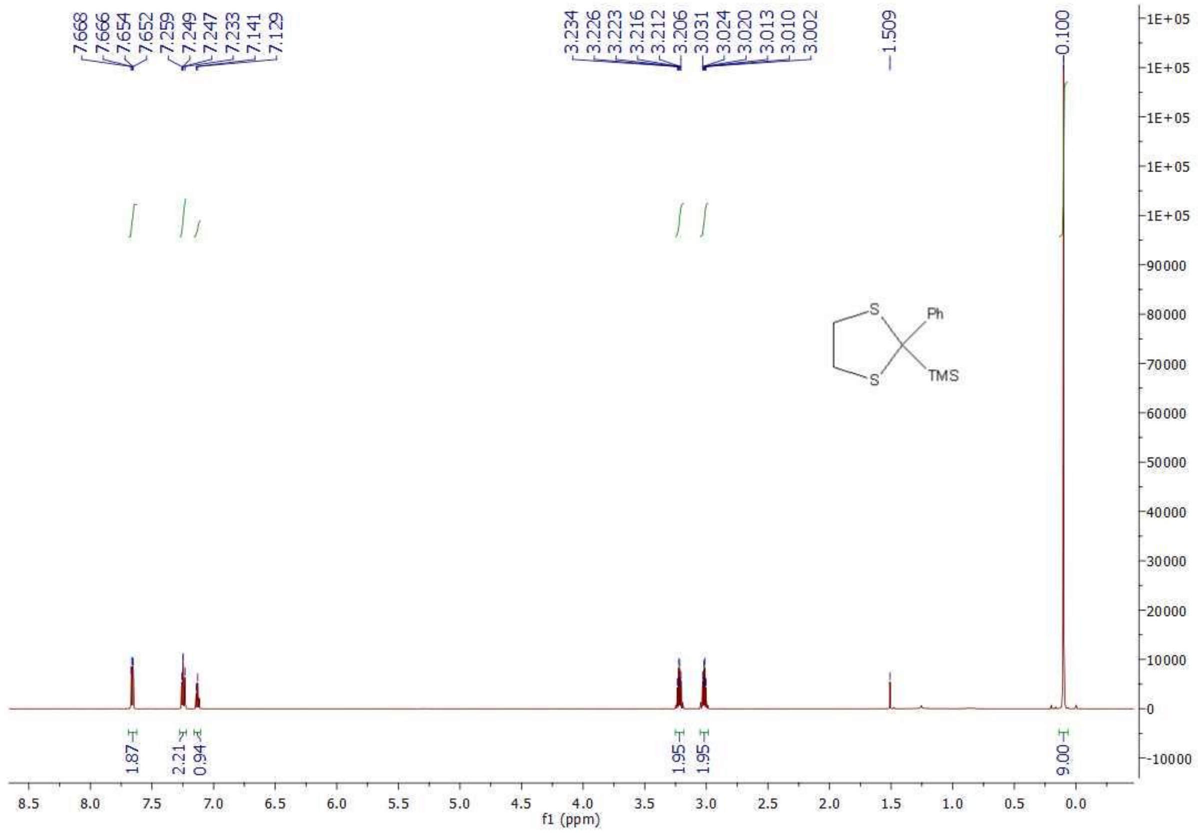

Figure S24. The ^{1}H NMR spectrum of compound **13b.**

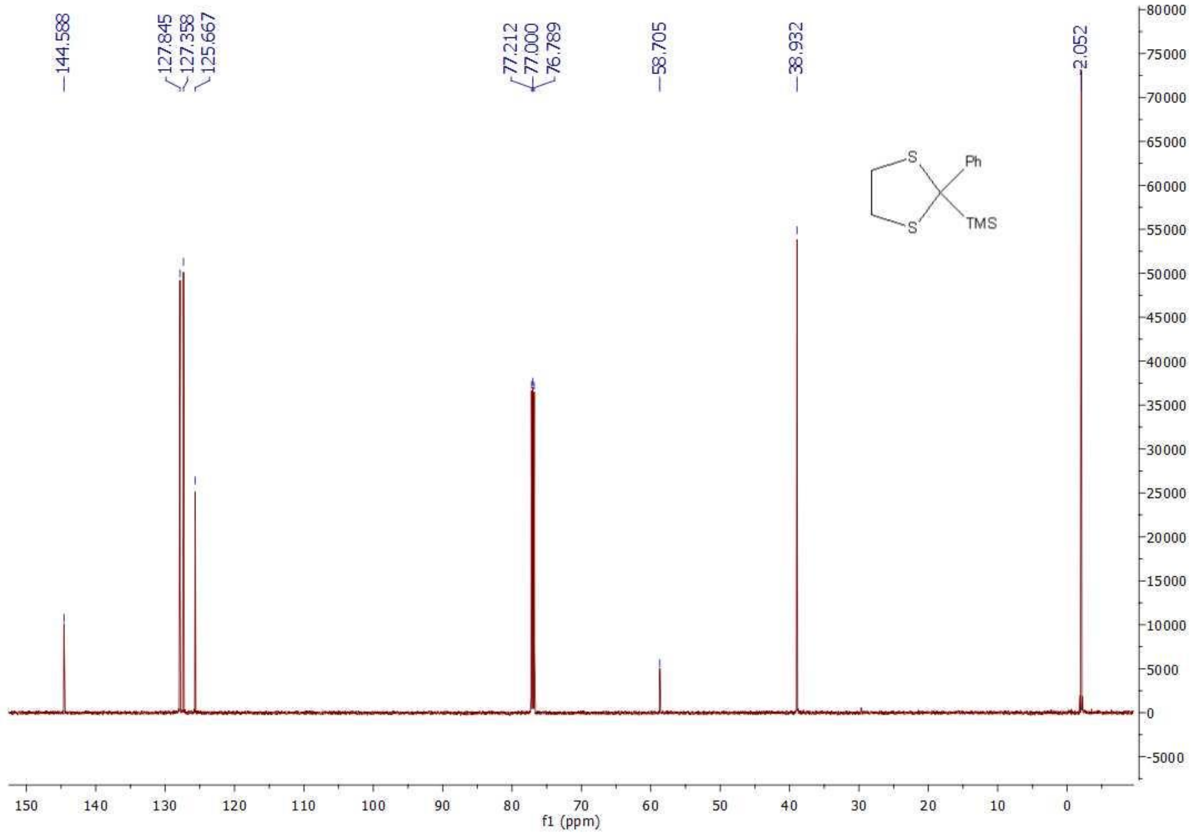

Figure S25. The ^{13}C NMR spectrum of compound **13b**.

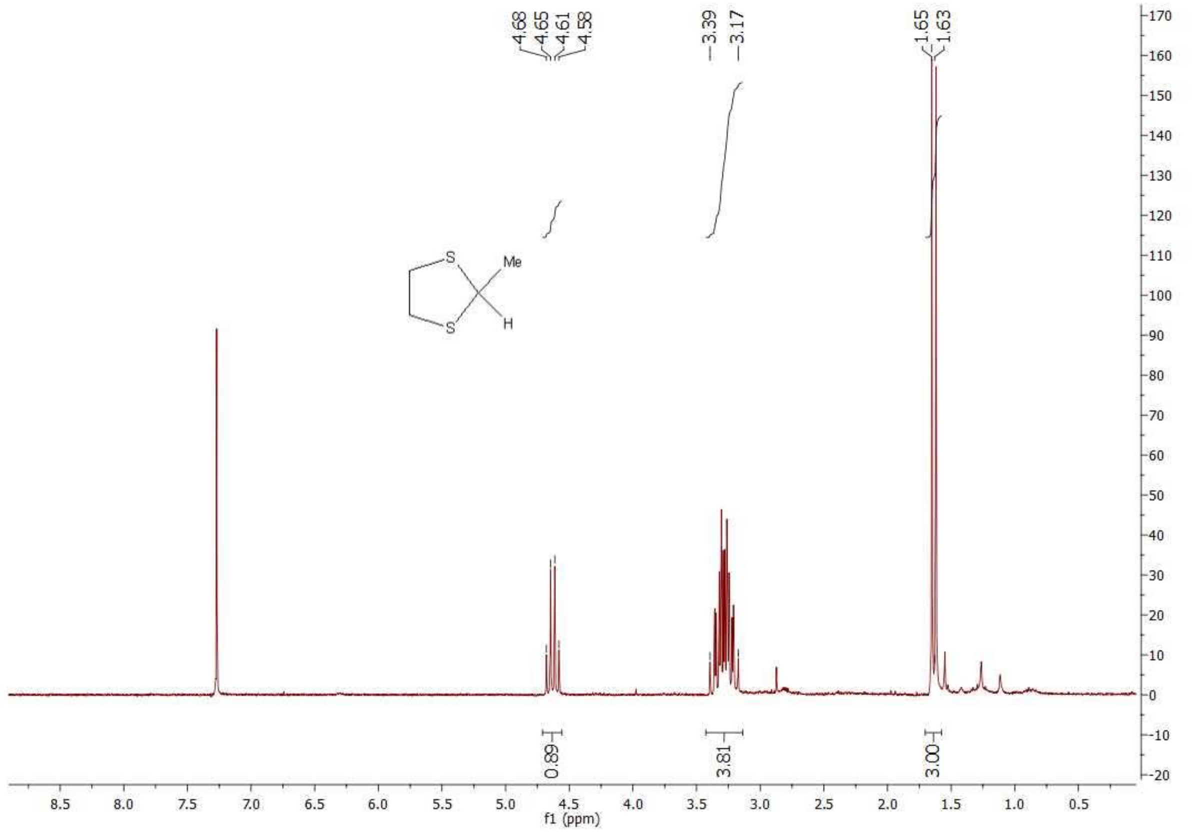

Figure S26. The ^{1}H NMR spectrum of compound **15a.**

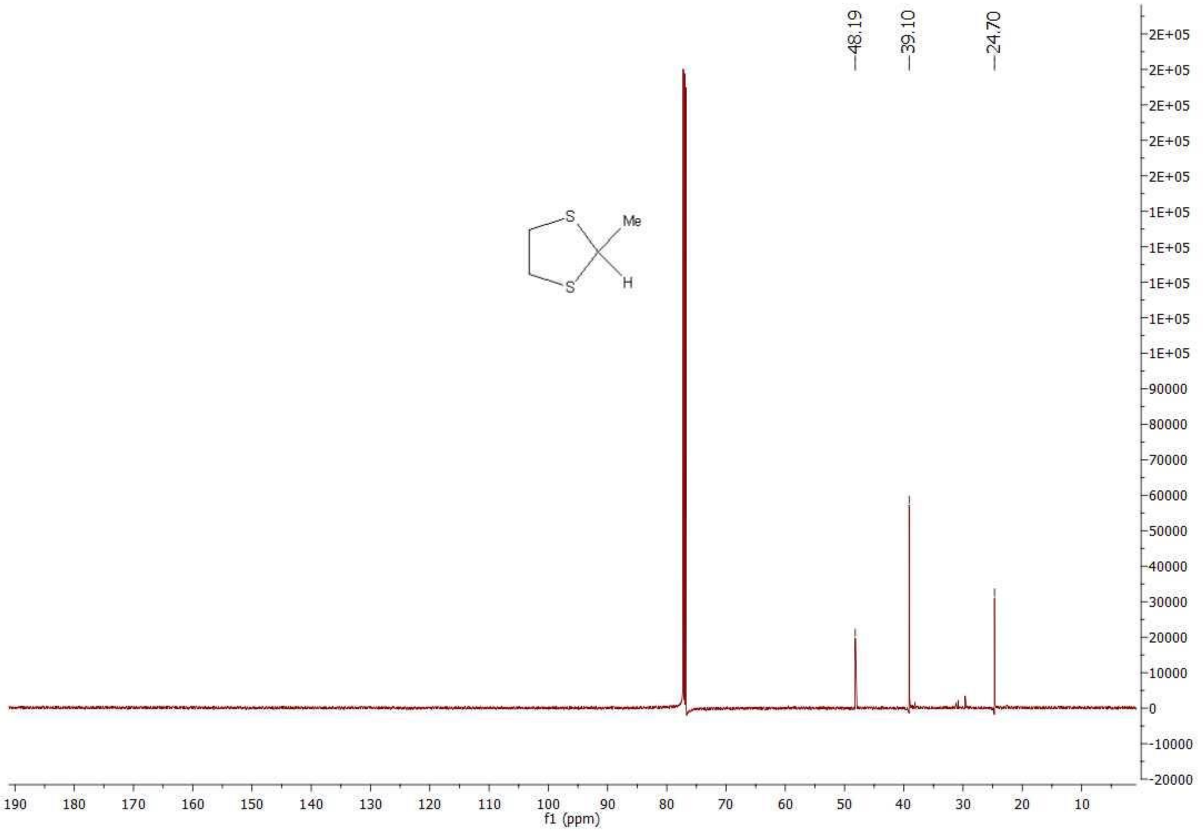

Figure S27. The ^{13}C NMR spectrum of compound **15a**.

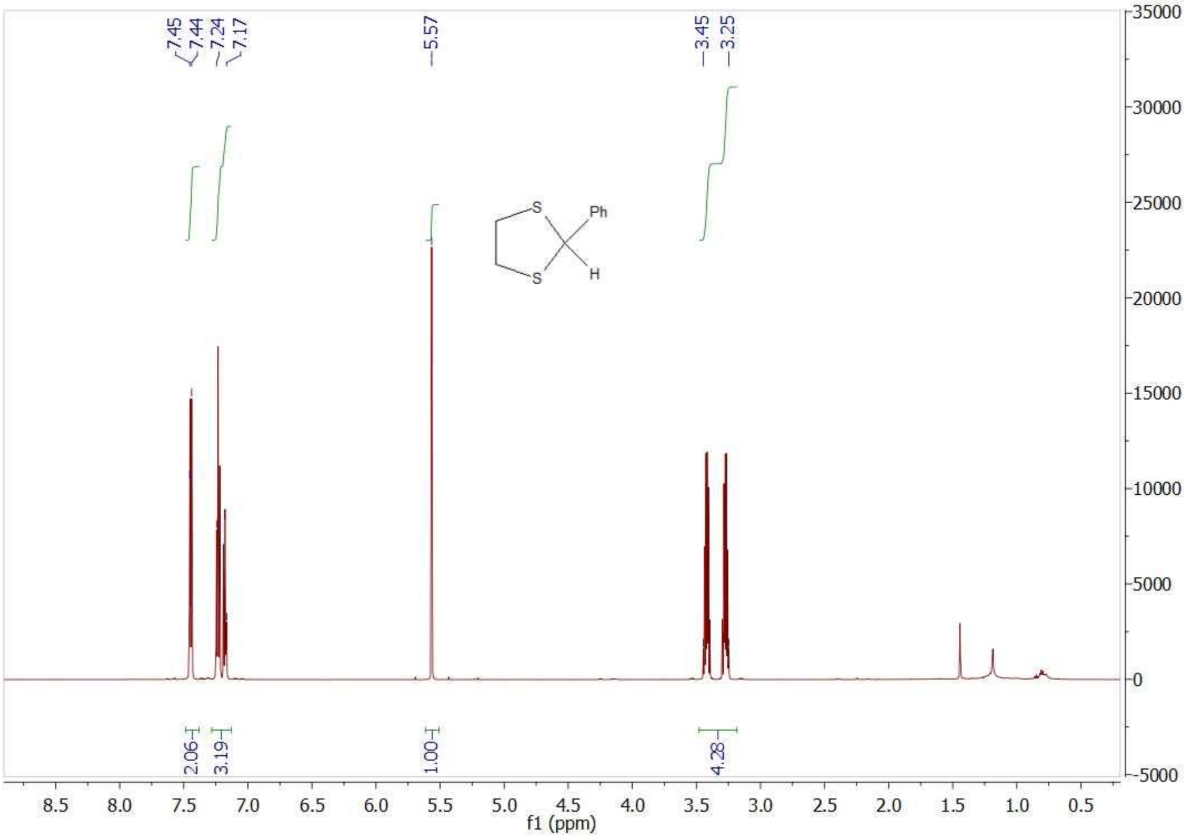

Figure S28. The ^{1}H NMR spectrum of compound **15b.**
